# Synthesis and structural characterisation of amides from picolinic acid and pyridine-2,6-dicarboxylic acid

**DOI:** 10.1038/srep09950

**Published:** 2015-05-08

**Authors:** Prarthana Devi, Sarah M. Barry, Kate M. Houlihan, Michael J. Murphy, Peter Turner, Paul Jensen, Peter J. Rutledge

**Affiliations:** 1 School of Chemistry F11, The University of Sydney, NSW 2006, Australia

## Abstract

Coupling picolinic acid (pyridine-2-carboxylic acid) and pyridine-2,6-dicarboxylic acid with *N*-alkylanilines affords a range of mono- and bis-amides in good to moderate yields. These amides are of interest for potential applications in catalysis, coordination chemistry and molecular devices. The reaction of picolinic acid with thionyl chloride to generate the acid chloride *in situ* leads not only to the *N*-alkyl-*N*-phenylpicolinamides as expected but also the corresponding 4-chloro-*N*-alkyl-*N*-phenylpicolinamides in the one pot. The two products are readily separated by column chromatography. Chlorinated products are not observed from the corresponding reactions of pyridine-2,6-dicarboxylic acid. X-Ray crystal structures for six of these compounds are described. These structures reveal a general preference for *cis* amide geometry in which the aromatic groups (*N*-phenyl and pyridyl) are *cis* to each other and the pyridine nitrogen *anti* to the carbonyl oxygen. Variable temperature ^1^H NMR experiments provide a window on amide bond isomerisation in solution.

Amides of general structures **1** and **2** (Figure 1) have a range of potential applications as ligands for catalysis, in molecular switches, and as metal binding agents. When combined with iron(II), ligands of this ilk can promote alkene dihydroxylation and allylic oxidation reactions akin to those mediated by non-heme iron oxidase enzymes (NHIOs)^1^,^2^,^3^,^4^,^5^,^6^,^7^,^8^,^9^; in combination with cobalt(III) or iron(III), they may catalyse conversion of nitriles to primary amide products, as mimics of the metalloenzyme nitrile hydratase^10^,^11^,^12^,^13^.

These compounds are of interest for potential application in molecular switches and devices that exploit the controlled *cis*/*trans* isomerisation of the amide bond^14^,^15^,^16^. They have demonstrated utility in coordination chemistry with transition metals^17^ and lanthanoids^18^, and have been applied to radionucleotide extraction^19^,^20^,^21^.

We report herein the synthesis of eleven amides (Figure 2) from picolinic acid **3** and pyridine-2,6-dicarboxylic acid **4**, and structural investigations using X-ray crystallography and variable temperature NMR.

## Results & Discussion

### Synthesis

Amides **5a–c**, **6a–c**, **7a–c**, **8a–b** were prepared by activating picolinic acid **3** and pyridine-2,6-dicarboxylic acid **4** to the corresponding acid chlorides *in situ*^22^, or via diimide-mediated peptide coupling^23^. Activating picolinic acid **3** with thionyl chloride afforded not only the simple picolinamides **5a–c** as expected, but also the 4-chloropicolinamides **6a–c** in the same pot. The two products were easily separated by column chromatography, enabling a ‘two for the price of one’ synthesis of new amides.

The mono-amide ligands **5a***–***c** and **6a***–***c** were synthesised from picolinic acid **3** and the corresponding aniline in one pot, via the acid chloride (Figure 3a). Thus acid **3** was treated with thionyl chloride overnight, followed by *N*-methylaniline, *N*-ethylaniline or *N*-diphenylamine and triethylamine in dichloromethane. This route gave the anticipated products **5a***–***c** in low to moderate yields (31–54%), and the 4-chloro derivatives **6a***–***c**, isolated in small but utilisable yields (10–13%). Each pair of compounds was readily separated by column chromatography.

Chlorination of the ring presumably occurs via activation of the pyridine to nucleophilic attack by chloride anion. This could occur during formation of the acid chloride or in the subsequent coupling step. The direct synthesis of 4-chloropicolinyl chloride from picolinic acid using thionyl chloride has been reported previously^24^,^25^, although in our own prior work we have converted picolinic acid to picolinoyl chloride with this reagent system, then reacted the acyl chloride with l-proline, without observing ring-chlorinated side products^7^. Our efforts to characterise the acid chloride intermediate(s) were unsuccessful: we were able to isolate a low-melting orange solid (mp ~ 40–50°C) but this quickly decomposed before it could be further characterised.

The *N*-methyl mono-amide **5a** has been prepared previously by Habib and Rees, who reported its synthesis, melting point and elemental analysis^26^, and more recently by Okamoto *et* *al.* as part of an investigation into acid-induced conformational changes in aromatic amides^14^. Habib and Rees prepared **5a** via the acid chloride, reacting picolinic acid **3** and thionyl chloride in benzene, then adding *N*-methylaniline dropwise and heating at reflux; Okamoto activated acid **3** as the mixed anhydride by reaction with ethyl chloroformate and triethylamine, before adding *N*-methylaniline. The 4-chloro derivative **6a** was not isolated in either of these previous syntheses.

Bis-amides **7a–c** were prepared in a similar manner, from pyridine-2,6-dicarboxylic acid **4** in one pot (Figure 3b). This gave compounds **7a–c** as crystalline solids in excellent yield (86–90%); chlorinated byproducts were not observed from the reactions of dicarboxylic acid **4**. Compounds **7a** and **7b** appear previously in the literature, but details of their synthesis and characterisation are incomplete. Ried and Neidhardt studied “hydrogenolysis” of the *N*-methyl compound **7a** and related quinoline carboxylic acids upon reaction with lithium aluminium hydride^27^. The *N*-methyl (**7a**) and *N*-ethyl (**7b**) analogues have been used to generate metal complexes^17^,^18^ and in metal extraction experiments^19^,^20^,^21^, while Dobler *et al.* conducted computational experiments to describe the interaction between ligands of this type and lanthanide cations^28^. Kapoor and coworkers recently reported synthesis and structural characterisation of related thioamide derivatives^29^.

In a complementary approach, the peptide derivatives **8a** and **8b** were prepared from pyridine-2,6-dicarboxylic acid **4** using diimide coupling methodology^23^. Thus dipeptides l-valinyl-*S*-benzyl-l-cysteine methyl ester (tosylate salt) **9** and *S*-benzyl-l-cysteinyl-l-valine methyl ester **10** (prepared from l-cysteine and l-valine via standard methods^30^) were coupled with **4** to give the peptide derivatives **8a** and **8b** in moderate yields (Figure 3c).

### Crystallographic investigations

The geometry of the amide bond in compounds such as these has received attention previously with a view to potential applications in molecular switches and devices^14^,^15^,^16^. *N*-Alkylation – specifically *N*-methylation – has been shown to induce a change from *trans*-preferential to *cis*-preferential amides (Figure 4).

Thus while the amide bond in benzanilide **11** (R = H) is *trans*, the corresponding bond in *N*-methylbenzanilide **12** (R = Me) is preferentially *cis*, both in the crystalline state and in solution^16^. Likewise crystallographic and NMR characterisation of **5a** reported by Okamoto *et al.* show that the two aromatic groups adopt a *cis* relationship in that compound too^14^. To investigate the geometry of the amides prepared in the current study, single crystal X-ray structures were determined for the mono-amides **5b** and **5c**, 4-chloro mono-amides **6b** and **6c**, and bisamides **7a** and **7c** (Figures 5 and 6; Supplementary Information).

The structures of the *N*-methyl (**7a**) and *N*-ethyl (**5b**, **6b**) compounds reveal *cis* amide geometry in all cases: the aromatic groups (*N*-phenyl and pyridyl) are *cis* to each other, and the methyl or ethyl substituent is *cis* to the carbonyl group. There is also a general preference for the pyridine nitrogen to sit *anti* to the carbonyl oxygen(s). Among the mono-amides, these groups are anticlinal in **5b** (the O–C–C–N dihedral angle is 123.9°), **6b** (126.5°) and **6c** (137.6°), but synclinal in **5c** (56.7°) (Figure 5). Of the bis-amide structures, the pyridine nitrogen is anticlinal to both carbonyls in the tetraphenyl compound **7c**: there are two inequivalent molecules of **7c** in the crystal structure, which exhibit dihedral angles around the bond in question of 141.6° and 131.9°/139.1° and 149.8° respectively. However in the dimethyl compound **7a**, the pyridine nitrogen is *anti* to one of the amide carbonyls (137.2°) but *syn* to the other (−57.2°), which – in combination with the two *cis* amide bonds – positions the two phenyl groups in close proximity and an edge-to-face arrangement (Figure 6).

### Variable temperature NMR experiments

In light of the recent work by Okamoto *et al.* using ^1^H NMR to follow *cis*/*trans* isomerisation in related aromatic amides^14^, we were interested to note evidence for slow conformational change in the ^1^H NMR spectra of compounds **7a–c**. The room temperature ^1^H NMR spectra of **7a–c** are generally poorly resolved with considerable line broadening (in contrast to the spectra of corresponding mono-amides **5a–c** in which equivalent line broadening is not observed – see Supplementary Information). Variable temperature ^1^H NMR data for the ethyl substituted ligand **7b** (Figure 7) show that signals resolve as the temperature is increased, confirming that the observed line broadening arises due to slow conversion between amide conformational isomers at room temperature. For example the signal at ~ 3.7 ppm, due to the methylene protons of the ethyl group, is a broad apparent singlet at 300 K but a clearly resolved quartet at 350 K (see inset in Figure 7).

### Conclusion

Amides derived from picolinic acid **3** and pyridine-2,6-dicarboxylic acid **4** have potential applications in catalysis, coordination chemistry and molecular switches. These compounds are readily prepared via the acid chloride or applying peptide coupling reagents. X-Ray crystal structures reveal that the generally preferred geometry of these amides positions the aromatic groups *cis* to each other and the pyridine nitrogen *anti* to the carbonyl oxygen. Variable temperature NMR experiments indicate slow *cis*/*trans* isomerisation in solution for the bis-amide series.

## Methods

### Amide synthesis

#### General procedure 1

Thionyl chloride (8.0 mL, 109 2 mmol) was added to picolinic acid **3** (1.00 g, 8.20 mmol) and the resulting suspension was refluxed for 16 h. The orange coloured solution was reduced *in vacuo* to give the acid chloride as a bright orange oil. The oil was dissolved in dry DCM (40 mL) and cooled to 0°C. A solution of *N*-alkylaniline (16.20 mmol) and triethylamine (2.20 mL, 16.20 mmol) in dry DCM (20 mL) was added via cannula. The resulting purple coloured solution was stirred at 0°C for 20 min and at room temperature for 16 h after which time the solution had become dark brown. The solution was washed with half-saturated aqueous ammonium chloride solution (2 × 12 mL), water (2 × 6 mL) and dried (Na_2_SO_4_), then concentrated *in vacuo*.

#### General procedure 2

Thionyl chloride (4.0 mL, 60 mmol) was added to 2,6-pyridinedicarboxylic acid **4** (0.50 g, 3.0 mmol) and the resulting suspension was refluxed under an argon atmosphere for 16 h to give a clear yellow solution. Excess thionyl chloride was removed *in vacuo* and the acid chloride was dissolved in dry CH_2_Cl_2 _(10 mL) and cooled to 0°C. A solution of *N*-alkylaniline (12.0 mmol) and triethylamine (0.84 mL, 6.0 mmol) in dry DCM (2.5 mL) was added via cannula. The resulting mixture was stirred at room temperature for 16 h during which time a white precipitate formed. The suspension was washed with half-saturated aqueous ammonium chloride solution (2 × 6 mL) and water (2 × 3 mL), then dried (Na_2_SO_4_) and concentrated *in vacuo*.

#### General procedure 3

Pyridine-2,6-dicarboxylic acid **4** (0.10–0.30 g, 1 eq.), dipeptide amine (as the free amine or tosylate salt, 2 eq.), 1-ethyl-3-(3-dimethylaminopropyl)carbodiimide (EDCI, 2 eq.) and 1-hydroxybenzotriazole (HOBt, 2 eq.) were dissolved in DCM (10–30 mL) and triethylamine (2 eq. for free amine, 4 eq. for tosylate salt) was added. The reaction mixture was stirred at room temperature for 22–48 h while monitored by TLC. Additional DCM or chloroform (10–20 mL) was added and the solution washed with equivalent volumes of water, 1 m hydrochloric acid, saturated sodium bicarbonate (aqueous) and brine, dried (MgSO_4_) then concentrated *in vacuo*.

#### N-Methyl-N-phenylpicolinamide **5a** and 4-Chloro-N-methyl-N-phenylpicolinamide **6a**

Picolinic acid **3** (1.0 g, 8.2 mmol) and *N*-methylaniline (1.76 mL, 16.2 mmol) were coupled using thionyl chloride (Procedure 1). TLC of the crude mixture showed the presence of two products, which were separated by flash column chromatography (petroleum benzine/ethyl acetate, 1:1) to afford **5a** (0.60 g, 35%) as a white crystalline solid and **6a** (0.27 g, 13%) as a thick, clear, colourless oil.

Data for *N*-methyl-*N*-phenylpicolinamide **5a** in agreement with literature^14^; see Supplementary Information for details.

Data for 4-chloro-*N*-methyl-*N*-phenylpicolinamide **6a**: R_f_ 0.40 (petroleum benzine/ethyl acetate, 1:1); ν_max _(CHCl_3_, cm^−1^) 3060 (w), 2997 (m), 1662 (s), 1581 (s), 1353(s), 1303(s); δ_H_ (400 MHz, (CD_3_)_2_CO) 3.43 (3H, s, NCH_3_), 6.97–7.19 (6H, m, NC_6_H_5_, 1 × pyr-CH), 7.45 (1Η, s, 1 × pyr-CH), 8.12 (1H, bs, 1 × pyr-CH); δ_C_ (100 MHz, (CD_3_)_2_CO) 38.0, 124.6, 124.9, 127.5, 127.9, 129.9, 144.7, 145.3, 150.5, 157.7, 167.9; *m/z* (ES+) 247 (65%, [MH]^+^ for ^35^Cl), 249 (20%, [MH]^+^ for ^37^Cl); HRMS (ES+) C_13_H_11_ClN_2_NaO^+^ ([M+Na]^+^ for ^35^Cl) requires 269.04540, found 269.04591.

#### N-Ethyl-N-phenylpicolinamide **5b** and 4-Chloro-N-ethyl-N-phenylpicolinamide **6b**

Picolinic acid **3** (1.0 g, 8.2 mmol) and *N*-ethylaniline (2.0 mL, 16.2 mmol) were coupled using thionyl chloride (Procedure 1). The crude product was purified by flash column chromatography (petroleum benzine/ethyl acetate, 1:1) to afford 5**b** (0.57 g, 31%) and **6b** (0.21 g, 10%) as white solid products.

Data for *N*-ethyl-*N*-phenylpicolinamide **5b**: R_f_ 0.30 (petroleum benzine/ethyl acetate, 1:1); mp: 87–92°C; ν_max _(CHCl_3_, cm^−1^) 3089, 3064 (w), 2950 (s) 1600 (s), 1492 (s), 1377 (s), 1272 (s); δ_H_ (400 MHz, (CD_3_)_2_CO) 1.17 (3Η, t, *J* = 7.0 Hz, NCH_2_CH_3_), 3.95 (2H, q, *J* = 7.0 Hz, NCH_2_CH_3_), 7.12−7.23 (6H, m, NC_6_H_5_, 1 × pyr-CH), 7.48 (1H, d, *J* = 7.5 Hz, 1 × pyr-CH), 7.70 (1H, bs, 1 × pyr-CH), 8.26 (1H, bs, 1 × pyr-CH); δ_C_ (100 MHz, (CD_3_)_2_CO) 12.3, 44.3, 123.2, 126.3, 128.0, 128.6, 136.1, 142.9, 148.0, 155.3, 167.8; *m/z* (ES+) 227 (26%, [MH]^+^), 249 (17%, [MNa]^+^), 475 (100%, [2MNa]^+^); HRMS (ES+) C_14_H_14_N_2_ONa^+^ ([M + Na]^+^) requires 249.09984, found 249.09967.

Data for 4-chloro-*N*-ethyl-*N*-phenylpicolinamide **6b**: R_f_ 0.40 (petroleum benzine/ethyl acetate, 1:1); mp: 80–83°C; ν_max _(CHCl_3_, cm^−1^) 3001 (w), 1650 (s), 1593 (s), 1554(m), 1492(m), 1311(m); δ_H_ (400 MHz, (CD_3_)_2_CO) 1.18 (3Η, t, *J* = 7.0 Hz, NCH_2_CH_3_), 3.93 (2H, q, *J* = 7.0 Hz, NCH_2_CH_3_), 7.17–7.29 (6H, m, NC_6_H_5_, 1 × pyr-CH), 7.57 (1H, s, 1 × pyr-CH), 8.22 (1H, bs, 1 × pyr-CH); δ_C_ (100 MHz, (CD_3_)_2_CO) 13.1, 45.3, 124.4, 127.6, 129.1, 129.7, 143.4, 144.5, 150.4, 157.8, 167.4; *m/z* (ES+) 261 (100%, [MH]^+^ for ^35^Cl), 263 (30%, [MH]^+^ for ^37^Cl); HRMS (ES+) C_14_H_14_ClN_2_O^+^ ([MH]^+^ for ^35^Cl) requires 261.07912, found 261.07938.

#### N,N-Diphenylpicolinamide **5c** and 4-Chloro-N,N-diphenylpicolinamide **6c**

Picolinic acid **3** (1.0 g, 8.2 mmol) and *N*-phenylaniline (2.74 g, 16.2 mmol) were coupled using thionyl chloride (Procedure 1). The crude product was purified by flash column chromatography (petroleum benzine/ethyl acetate, 1:1) to afford **5c** (1.21 g, 54%) and **6c** (0.25 g, 10%) as white solid products.

Data for *N*,*N*-diphenylpicolinamide **5c**: R_f_ 0.30 (petroleum benzine/ethyl acetate, 1:1); mp: 129–132°C; ν_max _(KBr, cm^−1^) 3058 (w), 1670 (s), 1587 (m), 1488 (m); δ_H_ (400 MHz, (CD_3_)_2_CO) 7.19–7.32 (10Η, m, N(C_6_H_5_)_2_), 7.63 (1H, d, *J* = 1.0 Hz, 1 × pyr-CH), 7.77 (1H, t, *J* = 7.5 Hz, 1 × pyr-CH), 8.28–8.30 (2H, m, pyr-CH); δ_C_ (100 MHz, (CD_3_)_2_CO) 124.5, 124.7, 126.9, 127.8, 129.4, 136.9, 143.8, 148.9, 154.9, 169.2; *m/z* (ES+) 275 (100%, [MH]^+^), 297 (55%, [MNa]^+^); HRMS (ES+) C_18_H_14_N_2_ONa^+^ ([M + Na]^+^) requires 297.09984, found 297.09958.

Data for 4-chloro-*N*,*N*-diphenylpicolinamide **6c**: R_f_ 0.40 (petroleum benzine/ethyl acetate, 1:1); mp: 122–124°C; ν_max _(CHCl_3_, cm^−1^) 3031 (m), 3024 (m), 1666 (s), 1643(s), 1593 (s), 1569(s) 1492 (s), 1404(s), 1350(s); δ_H_ (400 MHz, (CD_3_)_2_CO) 7.19−7.36 (11Η, m, N(C_6_H_5_)_2_, 1 × pyr-CH), 7.73 (1H, d, *J* = 2.0 Hz, 1 × pyr-CH), 8.27 (1H, d, *J* = 5.0 Hz, 1 × pyr-CH); δ_C_ (100 MHz, (CD_3_)_2_CO) 123.9, 124.1, 126.5, 127.6, 128.8, 143.3, 143.7, 149.6, 156.6, 166.9; *m/z* (ES+) 309 (90%, [MH]^+^ for ^35^Cl), 311 (35%, [MH]^+^ for ^37^Cl); HRMS (ES+) C_18_H_13_ClN_2_ONa^+^ ([M + Na]^+^ for ^35^Cl) requires 331.06111, found 331.06075.

#### N^2^, N^6^-Dimethyl-N^2^, N^6^-diphenylpyridine-2,6-dicarboxamide **7a**

2,6-Pyridinedicarboxylic acid **4** (0.50 g, 3.0 mmol) and *N*-methylaniline (1.28 mL, 12.0 mmol) were coupled using thionyl chloride (Procedure 2). The crude orange oil was triturated with hexane to yield the title compound as a white crystalline solid (0.89 g, 86%); R_f_ 0.20 (petroleum benzine/ethyl acetate, 1:1); mp: 148–155°C; ν_max _(KBr, cm^−1^) 3053 (w), 2969 (m), 2934 (w), 1651 (s), 1596 (m), 1585 (m); δ_H_ (400 MHz, (CD_3_)_2_CO) 3.31 (6H, s, 2 × NCH_3_), 7.04 (4H, br app. s, 4 of 2 × NC_6_H_5_), 7.16−7.30 (7H, m, 6 of 2 × NC_6_H_5_, 1 × pyr-CH), 7.66 (2H, br app s, 2 × pyr-CH); δ_C_ (100 MHz, (CD_3_)_2_CO) 37.2, 123.5, 126.3, 126.8, 128.9, 136.7, 144.5, 153.6, 167.3; *m/z* (ES+) 346 (73%, [MH]^+^), 368 (100%, [MNa]^+^); HRMS (ES+) C_21_H_20_N_3_O_2_^+^ ([MH]^+^) requires 346.15501, found 346.15501.

#### N^2^, N^6^-Diethyl-N^2^, N^6^-diphenylpyridine-2,6-dicarboxamide **7b**

2,6-Pyridinedicarboxylic acid **4** (0.50 g, 3.0 mmol) and *N*-ethylaniline (1.5 mL, 12.0 mmol) were coupled using thionyl chloride (Procedure 2). The crude product was purified by flash column chromatography (petroleum benzine/ethyl acetate, 1:1)to yield **7b** (0.99 g, 88%) as a white solid; R_f_ 0.20 (hexane/ether, 1:1); mp: 105–115°C; ν_max _(KBr, cm^−1^) 3055 (w), 2970 (m), 2931 (w), 1650 (s), 1596 (m), 1585 (m); δ_H_ (400 MHz, (CD_3_)_2_CO) 1.11 (6H, br app s, 2 × CH_2_CH_3_), 3.84 (4H, br app s, 2 × CH_2_CH_3_), 6.90–7.15 (4H, br app s, 4 of 2 × NC_6_H_5_), 7.16–7.35 (7H, m, 7 of 2 × NC_6_H_5_, 1 × pyr-CH), 7.58 (2H, br app s, 2 × pyr-CH); δ_C_ (100 MHz, (CD_3_)_2_CO) 13.3, 45.2, 124.2, 127.4, 129.0, 129. 7, 137.3, 143.6, 154.6, 167.7; *m/z* (ES+) 374 (50%, [MH]^+^), 396 (100%, [MNa]^+^); HRMS (ES+) C_23_H_24_N_3_O_2_^+^ ([MH]^+^) requires 374.18631, found 374.18631.

#### N^2^, N^2^, N^6^, N^6^-Tetraphenylpyridine-2,6-dicarboxamide **7c**

2,6-Pyridinedicarboxylic acid **4** (0.50 g, 3.0 mmol) and *N*-phenylaniline (2.05 g, 12.0 mmol) were coupled using thionyl chloride (Procedure 2). The crude product was purified by flash column chromatography (CH_2_Cl_2_/diethyl ether, 10:1) to give **7c** (1.26 g, 90%); R_f_ 0.25 (DCM/ether, 10:1); mp: 215–219°C; ν_max _(CHCl_3_, cm^−1^) 2999 (w), 1658 (s), 1639 (s), 1589 (s), 1485 (m), 1335 (s); δ_H_ (400 MHz, (CD_3_)_2_CO) 7.09 (8H, d, *J* = 7.5 Hz, 8 of 4 × NC_6_H_5_), 7.23–7.27 (4H, m, 4 of 4 × NC_6_H_5_), 7.32–7.36 (8H, m, 6 of 2 × NC_6_H_5_, 2 × pyr-CH), 7.58 (2H, d, *J* = 7.5 Hz, 2 of 2 × NC_6_H_5_), 7.77–7.81 (1H, m, 1 × pyr-CH); δ_C_ (100 MHz, (CD_3_)_2_CO) 125.4, 127.1, 128.5, 129.7, 137.9, 144.7, 153.8, 167.9; *m/z* (ES+) 470 (100%, [MH]^+^), 492 (43%, [MNa]^+^); HRMS (ES+) C_31_H_24_N_3_O_2_^+^ ([MH]^+^) requires 470.18631, found 470.18615.

#### Pyridine-2,6-dicarboxylic acid bis(l-valinyl-S-benzyl-l-cysteine methyl ester)carboxamide **8a**

2,6-Pyridinedicarboxylic acid **4** (0.10 g, 0.58 mmol) and l-valinyl-*S*-benzyl-l-cysteine methyl ester tosylate salt **9** (0.60 g, 1.2 mmol) were coupled using EDCI/HOBt (Procedure 3) to give **8a** as a yellow oil (0.20 g, 44%) after purification by column chromatography (cyclohexane/ethyl acetate, 1:4); R_f_ 0.65 (cyclohexane/ethyl acetate, 1:1); 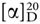 = + 1.5 (*c* = 2.0, CHCl_3_); ν_max_ (thin film) 3290 (s), 1745 (s), 1659 (s), 1530 (s); δ_H_ (300 MHz, CDCl_3_) 1.06 (12H, 2d, *J = * 6.5 Hz, 2 × CH(CH_3_)_2_), 2.25–2.37 (2H, m, 2 × CH(CH_3_)_2_), 2.86–2.88 (4H, m, 2 × CH_2_SCH_2_Ph), 3.66 (4H, s, 2 × SCH_2_Ph), 3.74 (6H, s, 2 × OCH_3_), 4.55 (2H, dd, *J = * 9.0, 7.0 Hz, 2 × CHNH), 4.80 (2H, dt, *J = * 7.5, 5.5 Hz, 2 × CHCH_2_S), 6.90 (2H, d, *J = * 7.5 Hz, 2 × NH), 7.17–7.70 (10H, m, 2 × C_6_H_5_), 7.88 (1H, m, 1 × pyr-CH), 8.34 (2H, d, *J = * 7.5 Hz, 2 × pyr-CH), 8.71 (2H, d, *J = * 9.0 Hz, 2 × NH); δ_C_ (75.4 MHz, CDCl_3_) 19.4, 28.4, 34.2, 36.6, 52.7, 58.9, 59.9, 127.3, 128.3, 128.7, 128.9, 137.6, 139.1, 148.6, 148.6, 163.7, 170.9; *m/z* (ES+) 780 (100%, [MH]^+^); HRMS (ES+) C_39_H_50_N_5_O_8_S_2_ ([MH]^+^) requires 780.3101, found 780.3112.

#### Pyridine-2,6-dicarboxylic acid bis(S-benzyl-l-cysteinyl-l-valine methyl ester)carboxamide **8b**

2,6-Pyridinedicarboxylic acid **4** (0.25 g, 1.5 mmol) and *S*-benzyl-l-cysteinyl-l-valine methyl ester **10** (1.20 g, 3.0 mmol) were coupled using EDCI/HOBt (Procedure 3) to give **8b** as a yellow oil (1.13 g, 61%), after purification by column chromatography (cyclohexane/ethyl acetate, 1:1); R_f_ 0.55 (cyclohexane/ethyl acetate 1:1); 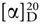 = -7.6 (*c* = 2.0, CHCl_3_); ν_max_ (thin film) 3420 (s, br), 3290 (s, br), 1740 (s), 1649 (s), 1538 (w); δ_H_ (300 MHz, CDCl_3_) 0.89 (12H, 2d, *J = * 4.5 Hz, 2 × CH(CH_3_)_2_), 2.11–2.22 (2H, m, 2 × CH(CH_3_)_2_), 2.91 (2H, dd, *J* = 14.0, 7.5 Hz, 2 of (2 × CH_2_S)), 3.01 (2H, dd, *J* = 14.0, 6.5 Hz, 2 of (2 × CH_2_S)), 3.74 (6H, s, 2 × OCH_3_), 3.84 (4H, s, 2 × SCH_2_Ph), 4.51 (2H, dd, *J = * 8.5, 5.0 Hz, 2 × CHNH), 4.69–4.77 (2H, m, 2 × CHCH_2_S), 6.94 (2H, d, *J = * 8.5 Hz, 2 × NH), 7.17–7.70 (10H, m, 2 × C_6_H_5_), 8.02–8.10 (2H, m, 2 × pyr-CH), 8.36 (1H, d, *J = * 7.5 Hz, 1 × pyr-CH), 8.83 (2H, d, *J = * 8.0 Hz, 2 × NH); δ_C_ (75.4 MHz, CDCl_3_) 17.8, 31.1, 33.4, 36.6, 52.2, 52.7, 57.6, 125.3, 127.2, 128.6, 129.1, 138.0, 138.6, 163.5, 163.6, 170.2, 171.9; *m/z* (ES+) 780 (50%, [MH]^+^); HRMS (ES+) C_39_H_50_N_5_O_8_S_2_ ([MH]^+^) requires 780.3101, found 780.3098.

## Author Contributions

**Figure 1:**
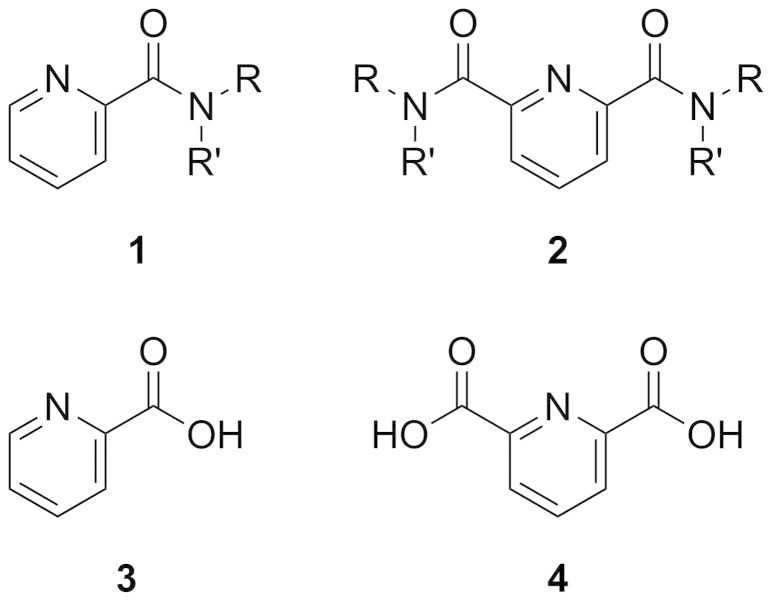
General structures of bidentate 1 and tridentate 2 amide targets, prepared from picolinic acid 3 and pyridine-2,6-dicarboxylic acid 4.

**Figure 2:**
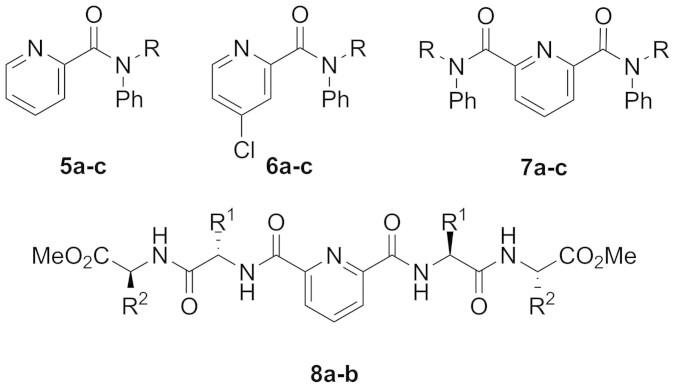
Structures of target amides 5–8. For **5a–7a** R = Me, **5b–7b** R = Et, **5c–7c** R = Ph; **8a** is derived from the l-valinyl-l-cysteine dipeptide (R^1^ = ^i^Pr, R^2^ = CH_2_SBn),</emph> while **8b** incorporates the l-cysteinyl-l-valine dipeptide (R^1^ = CH_2_SBn, R^2^ = ^i^Pr).

**Figure 3:**
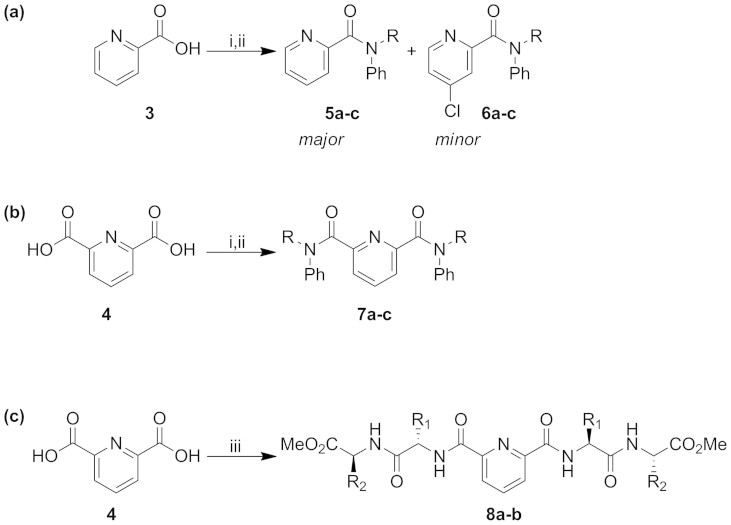
Synthesis of target compounds. (a) Synthesis of mono-amides **5a–c** and **6a–c**; i. SOCl_2_, reflux, 16 h; ii. Et_3_N, *N*-methylaniline a; *N*-ethylaniline b or *N*-diphenylamine c, DCM, rt, 16 h; **5a** 35%/**6a** 13%; **5b** 31%/**6b** 10%; **5c**. 54%/**6c** 10% (yields over two steps for major/minor products). (b) Synthesis of bis-amides **7a–c**; i. SOCl_2_, reflux, 16 h; ii. Et_3_N, *N*-methylaniline a, *N*-ethylaniline b or *N*-diphenylamine c (2 eq.), DCM, rt, 16 h; **7a** 86%, **7b** 88%, **7c** 90% (over two steps). (c) Synthesis of peptide derivatives **8a–b**; iii. EDCI, HOBt, Et_3_N, l-valinyl-*S*-benzyl-l-cysteine methyl ester tosylate salt **9** or *S*-benzyl-l-cysteinyl-l-valine methyl ester **10** (2 eq.), DCM, rt, 22–48 h; **8a** 44%, **8b** 61%.

**Figure 4:**
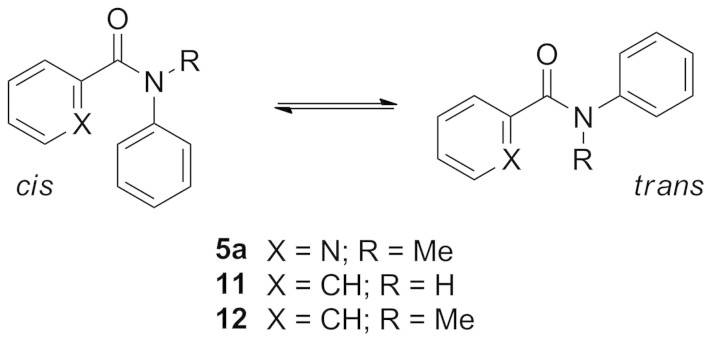
*Cis*/*trans* isomerisation in aromatic amides. While *trans* geometry is preferred when R = H, the *cis* isomer has been shown to predominate when R = Me^14^,^15^,^16^.

**Figure 5:**
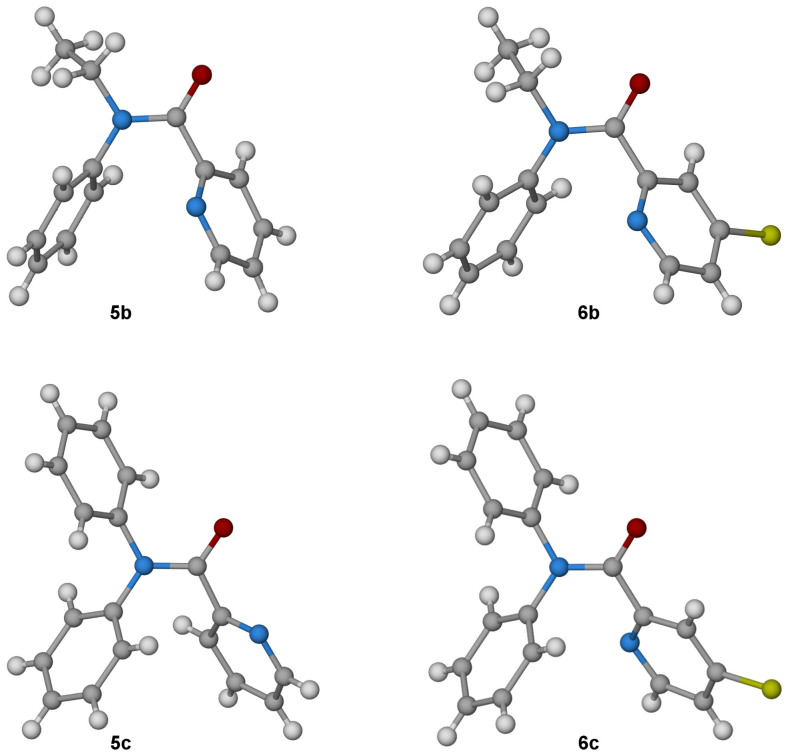
Crystal structures of amides 5b (CCDC-1002446), 5c (1002447), 6b (1002448), 6c (1002449). Carbon atoms are shown in grey, oxygen in red, nitrogen in blue and hydrogen in white. In **5b** and **6b**, the *cis* amide is observed, with the ethyl group *syn* to the carbonyl oxygen. In **5b**, **6b** and **6c** the pyridine nitrogen is *anti* to the carbonyl oxygen while in **5c** these atoms are *synclinal*.

**Figure 6:**
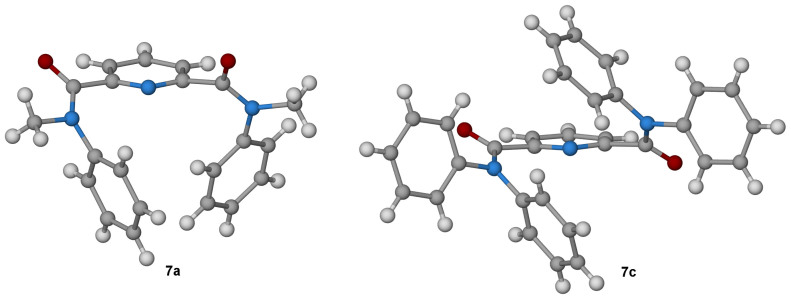
Crystal structures of amides 7a (CCDC-1002450) and 7c (1002451). Carbon atoms are shown in grey, oxygen in red, nitrogen in blue and hydrogen in white. In **7a** the methyl group and carbonyl oxygen are *cis*. The pyridine nitrogen is *anti* to both carbonyl oxygen atoms in **7c**, but *syn* to one and *anti* to the other in **7a**.

**Figure 7:**
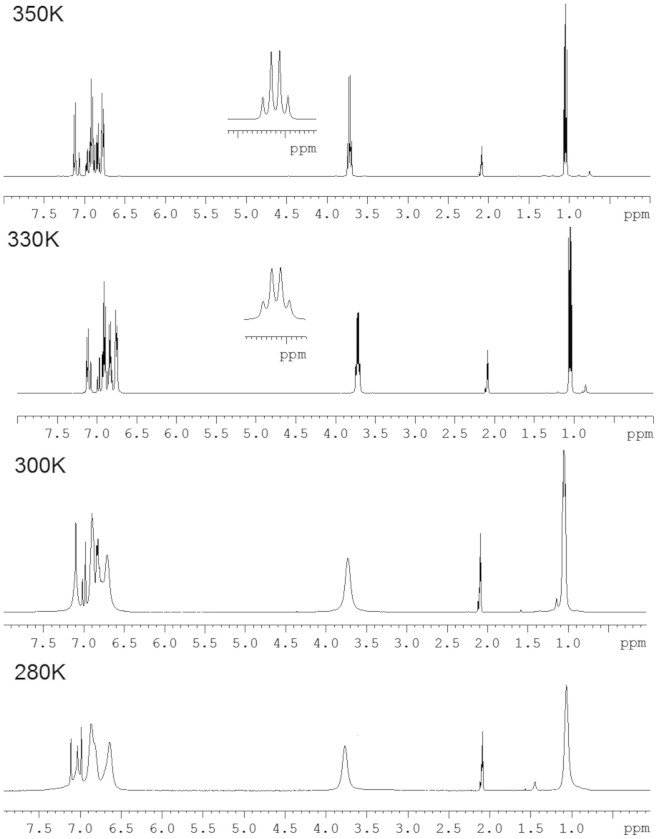
Variable temperature ^1^H NMR spectra of bis-amide ligand 7b (400 MHz, d_8_-toluene), confirming slow conformational change at room temperature.

S.M.B., K.M.H. and P.J.R. conceived and designed the experiments. P.D., S.M.B. and K.M.H. performed the synthetic experiments; M.J.M., P.T. and P.J. conducted X-ray crystallography experiments. M.J.M., P.T. and P.J. (crystallography), P.D., S.M.B., K.M.H. and P.J.R. analyzed the data. S.M.B. and P.J.R. wrote the main manuscript text including Figures 1–4 and 7; P.D. and M.J.M. prepared figures 5 and 6. All authors reviewed the manuscript.

## Supplementary Material

Supplementary Information Supplementary Information
