# Supplementary material for: Synthesis and structural characterisation of amides from picolinic acid and pyridine-2,6-dicarboxylic acid
Source: Sci Rep. 2015 May 8;5:9950. doi: 10.1038/srep09950 (PMC4424836; doi:10.1038/srep09950)

Supporting Information for

Synthesis and structural characterisation of amides from picolinic acid and pyridine-2,6-dicarboxylic acid.

Prarthana Devi, Sarah M. Barry,[†] Kate M. Houlihan, Michael J. Murphy, Peter Turner, Paul Jensen and Peter J. Rutledge[*]

School of Chemistry F11, The University of Sydney, NSW 2006, Australia.

[*] E-mail: peter.rutledge@sydney.edu.au; tel: +61 2 9351 5020; fax: +61 2 93513329

[†] *Current address:* Department of Chemistry, Faculty of Natural & Mathematical Sciences, King's College London, London, U.K.

Contents

1. General Experimental S1
2. Spectroscopic Data for *N*-Methyl-*N*-phenylpicolinamide **5a** S2
3. Crystallographic Experimental Details for Compounds **5b**, **5c**, **6b**, **6c**, **7a** and **7c** *S2*
4. References S6
5. ^{1}H and ^{13}C NMR spectra for compounds **5a–c**, **6a–c**, **7a–c**, and **8a–b** S8

1. *General Experimental*

All reaction solvents were distilled prior to use. Dry solvents were used where stated below. Methanol (MeOH) and acetonitrile (MeCN) were refluxed over CaH_2 and distilled under nitrogen immediately before use. Where stated below solvents were degassed using three freeze/thaw cycles. All reagents were obtained from chemical suppliers and used as received unless otherwise stated. *N*-Ethylaniline and *N*-methylaniline were distilled under vacuum from CaH_2 and stored under inert atmosphere over solid KOH. All alkenes used in turnover experiments were distilled over CaH_2 and stored over 4Å molecular sieves. Thin layer chromatography (TLC) was carried out on aluminium backed silica plates coated with Merck silica gel 60F254. TLC plates were viewed under ultra violet light (254 nm) or by staining with ammonium molybdate solution (10%w/v in 2 M sulphuric acid), ninhydrin (0.3% w/v in ^{n}BuOH:AcOH 97:3) for visualising amines or 'Goofy's stain' (0.01% Phosphomolybdic acid, 0.001% ceric sulphate, H_2O:H_2SO_4:EtOH 20:1:40) followed by heating. Retention factors are quoted to the nearest 0.05. Flash column chromatography was carried out using Ajax Finechem silica gel 230-400 mesh (40–63 microns).

Melting points were measured on a GallenKamp melting point apparatus. Infrared spectra were recorded on a Shimadzu FTIR8400S with Shimadzu IRSolution 1.04 software. Nuclear magnetic resonance spectra were recorded on Bruker Avance 200 MHz and Bruker Avance 400 MHz spectrometers. Variable temperature ^{1}H NMR was carried out on a Bruker Avance 400 MHz at the University of Sydney by Dr Ian Luck. Carbon spectra assignments are supported by DEPT editing where necessary. Low resolution mass spectrometry was carried out on a Finnigan LCQ MS Detector at the University of Sydney. High resolution mass spectrometry (HRMS) was carried out on a Bruker Apex II FTICR Electrospray. Mass spectra were recorded by Dr Keith Fisher.

2. Spectroscopic Data for N-Methyl-N-phenylpicolinamide 5a

This compound was prepared from picolinic acid **3** (1.0 g, 8.2 mmol) and *N*-methylaniline (1.66 mL, 16,2 mmol) using thionyl chloride (Procedure 1), and isolated as a white crystalline solid (0.60 g, 35 %); R_f 0.20 (petroleum benzine/ ethyl acetate, 1:1); ν_{max} ($CHCl_3$, cm^{-1}): 3087 (w), 2943 (w), 1660 (s), 1309(s); δ_H (400 MHz, $(CD_3)_2CO$) 3.43 (3H, s, $NC\underline{H}_3$), 7.12–7.22 (6H, m, $NC_6\underline{H}_5$, 1 × pyr-C$\underline{H}$), 7.50 (1H, d, *J* = 7.5 Hz, 1 × pyr-C$\underline{H}$), 7.72 (1H, t, *J* = 7.5, 1 × pyr-C$\underline{H}$), 8.27 (1H, bs, 1 × pyr-C$\underline{H}$); δ_C (100 MHz, $(CD_3)_2CO$) 37.0, 123.3, 123.7, 126.1, 126.8, 128.7, 136.2, 144.7, 148.0, 155.1, 168.2.; *m/z* (ES+): 213 (16 %, $[MH]^+$), 235 (25 %, $[MNa]^+$), 447 (100 %, $[2MNa]^+$); HRMS (ES+): $C_{13}H_{12}N_2ONa^+$ ($[M+Na]^+$) requires 235.08419, found 235.08442. Data in agreement with literature.[1]

3. Crystallographic Details for Compounds 5b, 5c, 6b, 6c, 7a and 7c

*Full supplementary crystallographic data for this paper can be obtained free of charge from The Cambridge Crystallographic Data Centre via www.ccdc.cam.ac.uk/data_request/cif under CCDC-1002446 (for **5b**), -1002447 (for **5c**), -1002448 (for **6b**), -1002449 (for **6c**), -1002450 (for **7a**) and -1002451 (for **7c**).*

N-Ethyl-N-phenylpicolinamide 5b

A colourless prismatic crystal was attached with Exxon Paratone N to a short length of fibre supported on a thin piece of copper wire inserted in a copper mounting pin. The crystal was quenched in a cold nitrogen gas stream from an Oxford Cryosystems Cryostream. A Bruker SMART 1000 X-ray diffractometer employing graphite monochromated Mo(Kα) radiation generated from a fine-focus sealed tube was used for the data collection. Cell constants were obtained from a least squares refinement against 6006 reflections located between 5 and 57° 2θ. Data were collected at 150(2) Kelvin with ω scans to 57° 2θ. The data integration and reduction were undertaken with SAINT and XPREP,[2] and subsequent computations were carried out with the X-Seed graphical user interface.[3] The intensities of 147 standard

reflections recollected at the end of the experiment did not change significantly during the data collection. An empirical absorption correction determined with SADABS was applied to the data.[4] The structure was solved in the space group *P*21/*n* (#14) by direct methods with SHELXS-97, and extended and refined with SHELXL-97.[5] The non-hydrogen atoms in the asymmetric unit were modelled with anisotropic displacement parameters. A riding atom model with group displacement parameters was used for the hydrogen atoms.

Summary Crystallographic Data: Formula $C_{14}H_{14}N_2O$, *M* 226.27, Monoclinic, space group $P2_1/n$(#14), *a* 9.1302(8), *b* 13.7960(13), *c* 9.9152(9) Å, β 106.174(2), *V* 1199.49(19) $Å^3$, D_c 1.253 g cm^{-3}, *Z* 4, crystal size 0.47 by 0.30 by 0.28 mm, colourless prisms, temperature 150(2) Kelvin, λ(MoKα) 0.71073 Å, μ(MoKα) 0.081 mm^{-1}, $T(SADABS)_{min,max}$ 0.934, 0.978, $2\theta_{max}$ 56.66, *hkl* range -11 11, -17 18, -12 13, *N* 11006, N_{ind} 2874(R_{merge} 0.0199), N_{obs} 2513(I > 2σ(I)), N_{var} 155, residuals $R1(F)$ 0.0362, $wR2(F^2)$ 0.0979, GoF(all) 1.046, $\Delta\rho_{min,max}$ -0.191, 0.289 e^- $Å^{-3}$. ($R1 = \Sigma||F_o| - |F_c||/\Sigma|F_o|$ for $F_o > 2\sigma(F_o)$; $wR2 = (\Sigma w(F_o^2 - F_c^2)^2/\Sigma(wF_c^2)^2)^{1/2}$ all reflections; $w=1/[\sigma^2(F_o^2)+(0.0475P)^2+0.2885P]$ where $P=(F_o^2+2F_c^2)/3$)

N,N-Diphenylpicolinamide 5c

A pale yellow prism-like crystal was attached with Exxon Paratone N, to a short length of fibre supported on a thin piece of copper wire inserted in a copper mounting pin. The crystal was quenched in a cold nitrogen gas stream from an Oxford Cryosystems Cryostream. A SuperNova (Dual Source) X-ray diffractometer equipped with an Atlas detector and employing mirror monochromated Cu (Kα) radiation from a micro-source was used for the data collection. Cell constants were obtained from a least squares refinement against 10388 reflections located between 10.16 and 151.88° 2θ. Data were collected at 150 Kelvin with ω scans to 152.46° 2θ. The data processing was undertaken with CrysAlis Pro[6] and subsequent computations were carried out with WinGX[7] and ShelXle.[8] A multi-scan absorption correction was applied to the data.[6] The structure was solved in the space group *Pbca* (#61) by direct methods with SHELXS-97,[5] and refined with SHELXL-97.[5] The non-hydrogen atoms in the asymmetric unit were modelled with anisotropic displacement parameters. A riding atom model with group displacement parameters was used for the hydrogen atoms.

Summary Crystallographic Data: Formula $C_{18}H_{14}N_2O$, *M* 274.31, orthorhombic, space group *P*bca (#61), *a* 17.2376(2), *b* 9.29600(10), *c* 17.3984(2) Å, *V* 2787.93(5) $Å^3$, D_c 1.307 g cm^{-3}, *Z* 8, crystal size 0.05 by 0.03 by 0.02 mm, colour pale yellow, habit prism, temperature 150(2) Kelvin, λ(CuKα) 1.54184 Å, μ(CuKα) 0.653 mm^{-1}, $T_{min,max}$ 0.87575, 1.00000, $2\theta_{max}$ 152.46, *hkl* range -19 21, -11 11, -20 21, *N* 22802, N_{ind} 2902(R_{merge} 0.0563), N_{obs} 2475(I > 2σ(I)), N_{var} 190, residuals* $R1(F)$ 0.0489, $wR2(F^2)$ 0.1156, GoF(all) 1.213, $\Delta\rho_{min,max}$ -0.214, 0.207 e^- $Å^{-3}$. ($R1 = \Sigma||F_o| - |F_c||/\Sigma|F_o|$ for $F_o > 2\sigma(F_o)$; $wR2 = (\Sigma w(F_o^2 - F_c^2)^2/\Sigma(wF_c^2)^2)^{1/2}$ all reflections; $w=1/[\sigma^2(F_o^2)+(0.0371P)^2+1.5257P]$ where $P=(F_o^2+2F_c^2)/3$)

4-Chloro-N-ethyl-N-phenylpicolinamide 6b

A pale yellow prism-like crystal was attached with Exxon Paratone N, to a short length of fibre supported on a thin piece of copper wire inserted in a copper mounting pin. The crystal was quenched in a cold nitrogen gas stream from an Oxford Cryosystems Cryostream. A SuperNova (Dual Source) X-ray diffractometer equipped with an Atlas detector and employing mirror monochromated Cu (Kα) radiation from a micro-source was used for the data collection cell constants were obtained from a least squares refinement against 7167 reflections located between 10.05 and 151.97° 2θ. Data were collected at 150 Kelvin with ω scans to 152.28° 2θ. The data processing was undertaken with CrysAlis Pro[6] and subsequent computations were carried out with WinGX[7] and ShelXle.[8] A multi-scan absorption correction was applied to the data.[6] The structure was solved in the space group *P*1 (#2) by direct methods with SHELXS-97,[5] and refined with SHELXL-97.[5] The non-hydrogen atoms in the asymmetric unit were modelled with anisotropic displacement parameters. A riding atom model with group displacement parameters was used for the hydrogen atoms.

Summary Crystallographic Data: Formula $C_{14}H_{13}ClN_2O$, *M* 260.71, triclinic, space group *P*1(#2), a 8.2033(4), b 9.2381(5), c 9.5518(4) Å, α 109.623(4), β 97.289(4), γ 105.203(4)°, V 639.30(5) Å^3, D_c 1.354 g cm^{-3}, Z 2, crystal size 0.07 by 0.06 by 0.05 mm, colour yellow, habit prismatic, temperature 150(2) Kelvin, λ(CuKα) 1.54178 Å, μ(CuKα) 2.552 mm^{-1}, $T_{min,max}$ 0.78321, 1.00000, $2\theta_{max}$ 152.28, *hkl* range -10 10, -11 10, -11 11, N 10970, N_{ind} 2655(R_{merge} 0.0223), N_{obs} 2507($I > 2\sigma(I)$), N_{var} 163, residuals $R1(F)$ 0.0313, $wR2(F^2)$ 0.0832, GoF(all) 1.064, $\Delta\rho_{min,max}$ -0.299, 0.258 e$^-$ Å^{-3}. ($R1 = \Sigma||F_o| - |F_c||/\Sigma|F_o|$ for $F_o > 2\sigma(F_o)$; $wR2 = (\Sigma w(F_o^2 - F_c^2)^2/\Sigma(wF_c^2)^2)^{1/2}$ all reflections; $w=1/[\sigma^2(F_o^2)+(0.0428P)^2+0.1963P]$ where $P=(F_o^2+2F_c^2)/3$).

4-Chloro-N,N-diphenylpicolinamide 6c

A colourless rod-like crystal was attached with Exxon Paratone N, to a short length of fibre supported on a thin piece of copper wire inserted in a copper mounting pin. The crystal was quenched in a cold nitrogen gas stream from an Oxford Cryosystems Cryostream. A SuperNova (Dual Source) X-ray diffractometer equipped with an Atlas detector and employing mirror monochromated Cu (Kα) radiation from a micro-source was used for the data collection cell constants were obtained from a least squares refinement against 16007 reflections located between 7.26 and 152.15° 2θ. Data were collected at 150 Kelvin with ω scans to 152.48° 2θ. The data processing was undertaken with CrysAlis Pro[6] and subsequent computations were carried out with WinGX[7] and ShelXle.[8] A multi-scan absorption correction was applied to the data.[6] The structure was solved in the space group *I4* (#82) by direct methods with SHELXS-97,[5] and refined with SHELXL-97.[5] The non-hydrogen atoms in the asymmetric unit were modelled with anisotropic displacement parameters. A riding atom model with group

displacement parameters was used for the hydrogen atoms. The absolute structure was established with the Flack parameter[9-12] refining to 0.000(12).

Summary Crystallographic Data: Formula $C_{18}H_{13}ClN_2O$, *M* 308.75, tetragonal, space group *I*4(#82), *a* 24.22260(10), *b* 24.22260(10), *c* 5.23930(10) Å, *V* 3074.08(6) Å^3, D_c 1.334 g cm^{-3}, *Z* 8, crystal size 0.11 by 0.035 by 0.025 mm, colour colourless, habit rod, temperature 150(2) Kelvin, λ(CuKα) 1.54178 Å, μ(CuKα) 2.216 mm^{-1}, $T_{min,max}$ 0.71170, 1.00000, $2\theta_{max}$ 152.48, *hkl* range -30 30, -30 30, -6 6, *N* 33711, N_{ind} 3221(R_{merge} 0.0393), N_{obs} 3124(I > 2σ(I)), N_{var} 200, residuals *R*1(*F*) 0.0300, *wR*2(F^2) 0.1053, GoF(all) 0.966, $\Delta\rho_{min,max}$ -0.248, 0.239 e$^-$ Å^{-3}. ($R1 = \Sigma||F_o| - |F_c||/\Sigma|F_o|$ for $F_o > 2\sigma(F_o)$; $wR2 = (\Sigma w(F_o^2 - F_c^2)^2/\Sigma(wF_c^2)^2)^{1/2}$ all reflections; $w=1/[\sigma^2(F_o^2)+(0.1000P)^2+0.0000P]$ where $P=(F_o^2+2F_c^2)/3$)

***N²*, *N⁶*-Dimethyl-*N²*, *N⁶*-diphenylpyridine-2,6-dicarboxamide 7a**

A pale yellow prism-like crystal was attached with Exxon Paratone N, to a short length of fibre supported on a thin piece of copper wire inserted in a copper mounting pin. The crystal was quenched in a cold nitrogen gas stream from an Oxford Cryosystems Cryostream. A SuperNova (Dual Source) X-ray diffractometer equipped with an Atlas detector and employing mirror monochromated Cu (Kα) radiation from a micro-source was used for the data collection cell constants were obtained from a least squares refinement against 2271 reflections located between 10.13 and 83.43° 2θ. Data were collected at 150 Kelvin with ω scans to 150.52° 2θ. The data processing was undertaken with CrysAlis Pro[6] and subsequent computations were carried out with WinGX[7] and ShelXle.[8] A multi-scan absorption correction was applied to the data.[6] The structure was solved in the space group *Pbca* (#61) by direct methods with SHELXS-97,[5] and refined with SHELXL-97.[5] The non-hydrogen atoms in the asymmetric unit were modelled with anisotropic displacement parameters. A riding atom model with group displacement parameters was used for the hydrogen atoms.

Summary Crystallographic Data: Formula $C_{21}H_{19}N_3O_2$, *M* 345.39, orthorhombic, space group *Pbca*(#61), *a* 8.2707(4), *b* 12.1599(11), *c* 34.963(2) Å, *V* 3516.3(4) Å^3, D_c 1.305 g cm^{-3}, *Z* 8, crystal size 0.223 by 0.165 by 0.118 mm, colour colourless, habit prism, temperature 150(2) Kelvin, λ(CuKα) 1.54178 Å, μ(CuKα) 0.689 mm^{-1}, $T_{min,max}$ 0.88359, 1.00000, $2\theta_{max}$ 150.52, *hkl* range -10 10, -15 15, -43 43, *N* 28963, N_{ind} 3602(R_{merge} 0.0582), N_{obs} 3464(I > 2σ(I)), N_{var} 237, residuals *R*1(*F*) 0.0382, *wR*2(F^2) 0.1099, GoF(all) 1.002, $\Delta\rho_{min,max}$ -0.236, 0.199 e$^-$ Å^{-3}. ($R1 = \Sigma||F_o| - |F_c||/\Sigma|F_o|$ for $F_o > 2\sigma(F_o)$; $wR2 = (\Sigma w(F_o^2 - F_c^2)^2/\Sigma(wF_c^2)^2)^{1/2}$ all reflections; $w=1/[\sigma^2(F_o^2)+(0.0700P)^2+1.0000P]$ where $P=(F_o^2+2F_c^2)/3$)

N^2,N^2,N^6,N^6-Tetraphenylpyridine-2,6-dicarboxamide 7c

A colourless prism like crystal was attached with Exxon Paratone N, to a short length of fibre supported on a thin piece of copper wire inserted in a copper mounting pin, and mounted on an APEXII-FR591 diffractometer employing mirror monochromated MoKα radiation generated from a rotating anode. Cell constants were obtained from a least squares refinement against 14375 reflections located between 6.20 and 59.06° 2θ. Data were collected at 150(2) Kelvin with ω+ϕ scans to 52.75° 2θ. The data integration and reduction were undertaken with SAINT and XPREP.[2] An empirical absorption correction determined with SADABS[4] was applied to the data. The structure was solved in the space group P1(#2) by direct methods with SIR97,[13] and extended and refined with SHELXL-13.[14] The atoms in the asymmetric unit were modelled with anisotropic displacement parameters. No hydrogen atoms were included in the model.

Summary Crystallographic Data: Formula $C_{31}H_{23}N_3O_2$, *M* 469.52, triclinic, space group P1(#2), *a* 9.3861(5), *b* 15.6791(10), *c* 17.8929(12) Å, α 71.624(4), β 83.341(3), γ 78.423(3)°, *V* 2444.2(3) Å^3, D_c 1.265 g cm^{-3}, *Z* 4, crystal size 0.142 by 0.113 by 0.054 mm, colour colourless, habit prism, temperature 150(2) Kelvin, λ(MoKα) 0.71073 Å, μ(MoKα) 0.081 mm^{-1}, T(SADABS)$_{min,max}$ 0.878, 1.000, $2\theta_{max}$ 52.75, *hkl* range -11 11, -19 19, -22 22, *N* 84637, N_{ind} 10008(R_{merge} 0.0682), N_{obs} 7101(I > 2σ(I)), N_{var} 649, residuals $R1(F)$ 0.0424, $wR2(F^2)$ 0.1337, GoF(all) 1.111, $\Delta\rho_{min,max}$ -0.393, 0.293 e$^-$ Å^{-3}. ($R1 = \Sigma||F_o| - |F_c||/\Sigma|F_o|$ for $F_o > 2\sigma(F_o)$; $wR2 = (\Sigma w(F_o^2 - F_c^2)^2/\Sigma(wF_c^2)^2)^{1/2}$ all reflections; $w=1/[\sigma^2(F_o^2)+(0.0500P)^2+1.5000P]$ where $P=(F_o^2+2F_c^2)/3$)

4. References

1 Okamoto, I., et al. Acid-induced conformational alteration of cis-preferential aromatic amides bearing N-methyl-N-(2-pyridyl) moiety. *Tetrahedron* **67**, 8536–8543 (2011).

2 Bruker. (Bruker Analytical X-ray Instruments Inc, Madison, Wisconsin, USA. , 1995).

3 Barbour, L.J. X-Seed - A software tool for supramolecular crystallography. *J. Supramol. Chem.* **1**, 189–191 (2001).

4 Sheldrick, G.M. (University of Göttingen, Göttingen, Germany, 1996).

5 Sheldrick, G.M. (University of Göttingen, Göttingen, Germany, 1998).

6 Agilent Technologies (Yarnton, Oxfordshire, UK, 2013).

7 Farrugia, L. WinGX suite for small-molecule single-crystal crystallography. *J. Appl. Crystallogr.* **32**, 837–838 (1999).

8 Hübschle, C.B. & Sheldrick, G.M.D., B. ShelXle: graphical user interface for SHELXL. *J. Appl. Cryst.* **44**, 1281–1284 (2011).

9 Flack, H.D. On enantiomorph-polarity estimation. *Acta Crystallogr., Sect. A: Found. Crystallogr.* **39**, 876–881 (1983).

10 Bernardinelli, G. & Flack, H.D. Least-squares absolute-structure refinement – practical experience and ancillary calculations. *Acta Crystallogr., Sect. A: Found. Crystallogr.* **41**, 500–511 (1985).

11 Flack, H.D. & Bernardinelli, G. Absolute structure and absolute configuration. *Acta Crystallogr., Sect. A: Found. Crystallogr.* **55**, 908–915 (1999).

12 Flack, H.D. & Bernardinelli, G. Reporting and evaluating absolute-structure and absolute-configuration determinations. *J. Appl. Crystallogr.* **33**, 1143–1148 (2000).

13 Altomare, A., et al. SIR97: a new tool for crystal structure determination and refinement. *J. Appl. Cryst.* **32**, 115–119 (1999).

14 Sheldrick, G.M. A short history of SHELX. *Acta Crystallogr., Sect. A: Found. Crystallogr* **64**, 112–122 (2008).

5. ***^{1}H and ^{13}C NMR spectra***

^{1}H NMR (400MHz, CD_3COCD_3) of N-methyl-N-phenylpicolinamide ***5a***

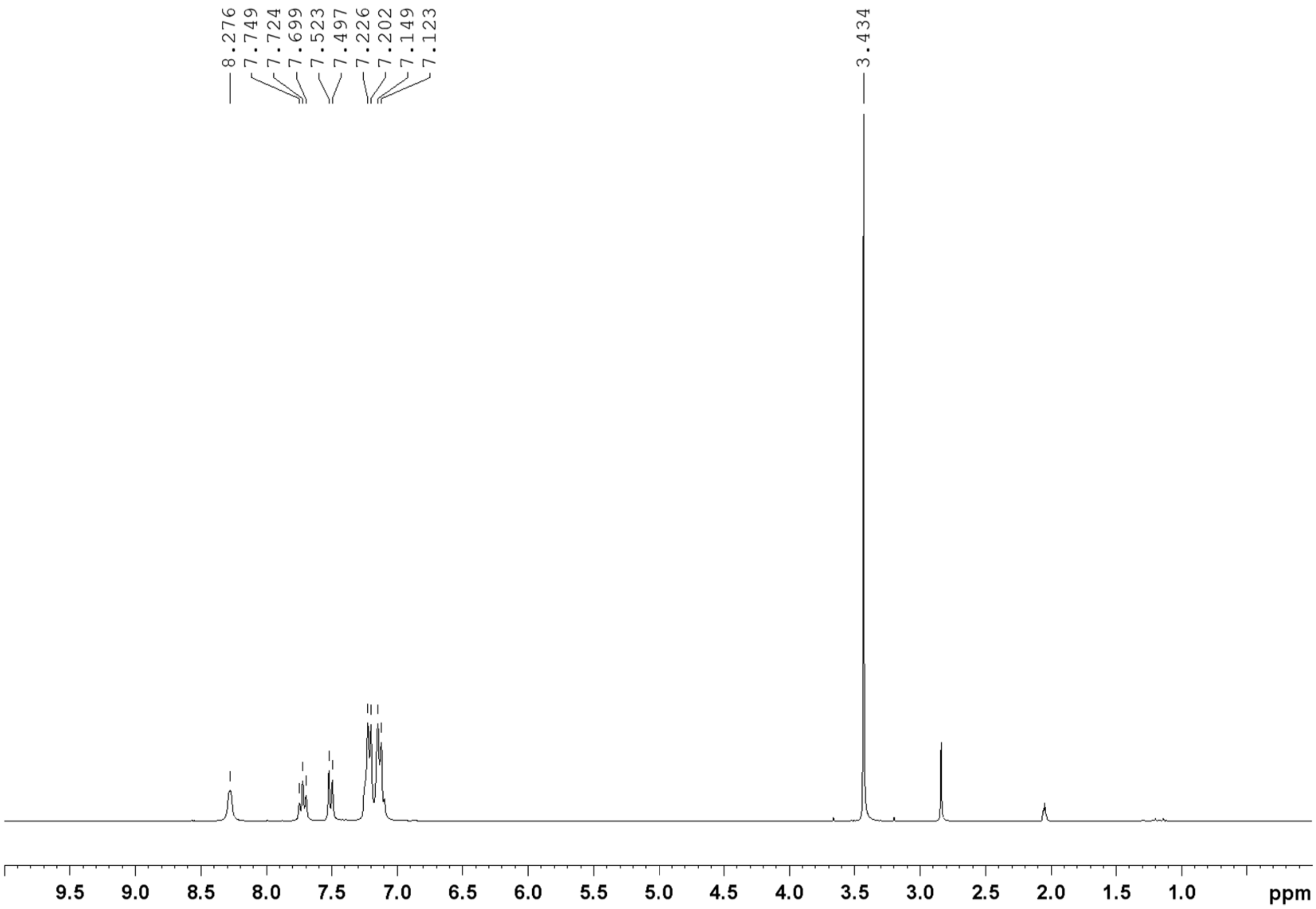

^{13}C NMR (100MHz, CD_3COCD_3) of N-methyl-N-phenylpicolinamide ***5a***

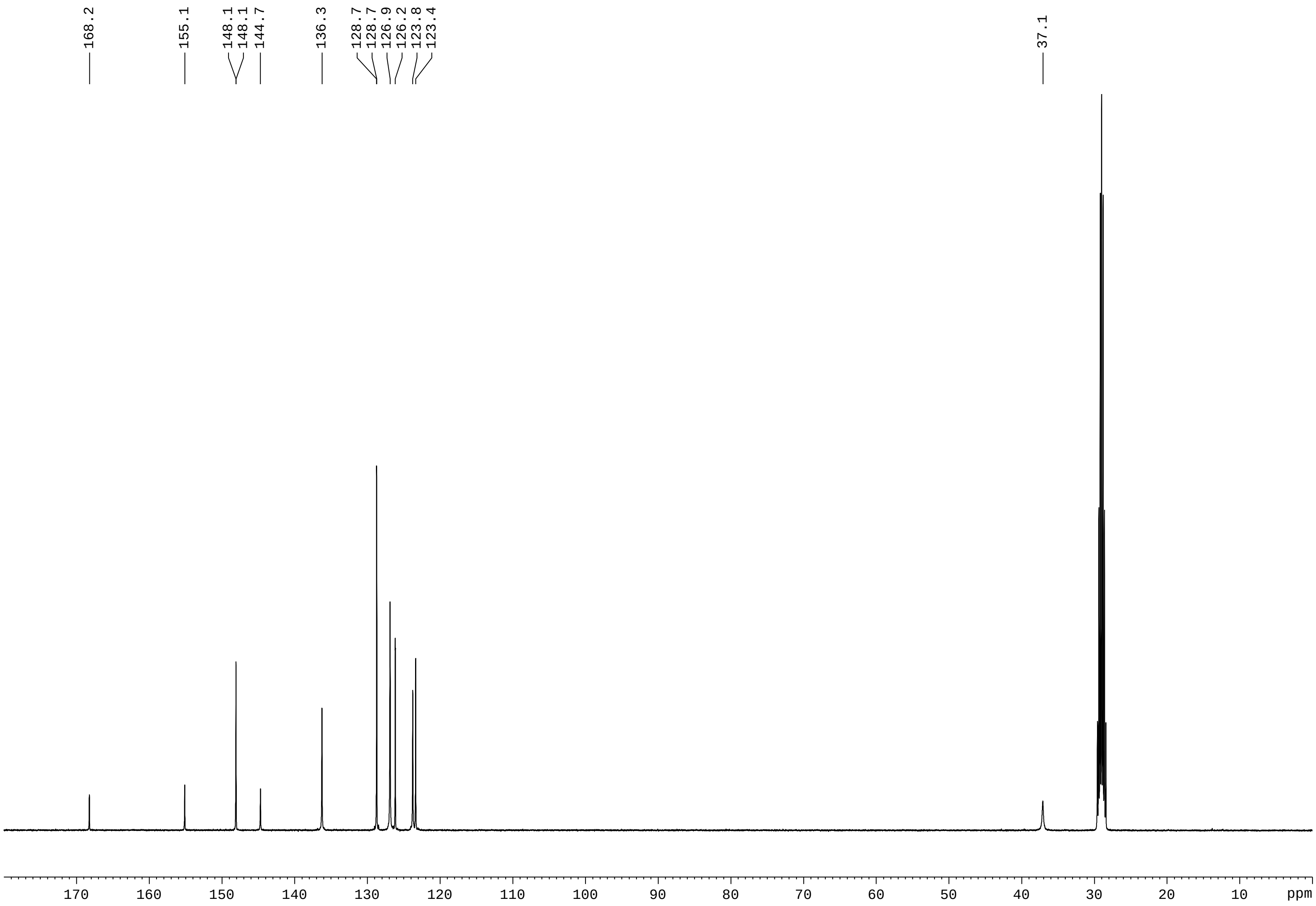

^{1}*H NMR (400MHz, CD_3COCD_3) of N-ethyl-N-phenylpicolinamide* ***5b***

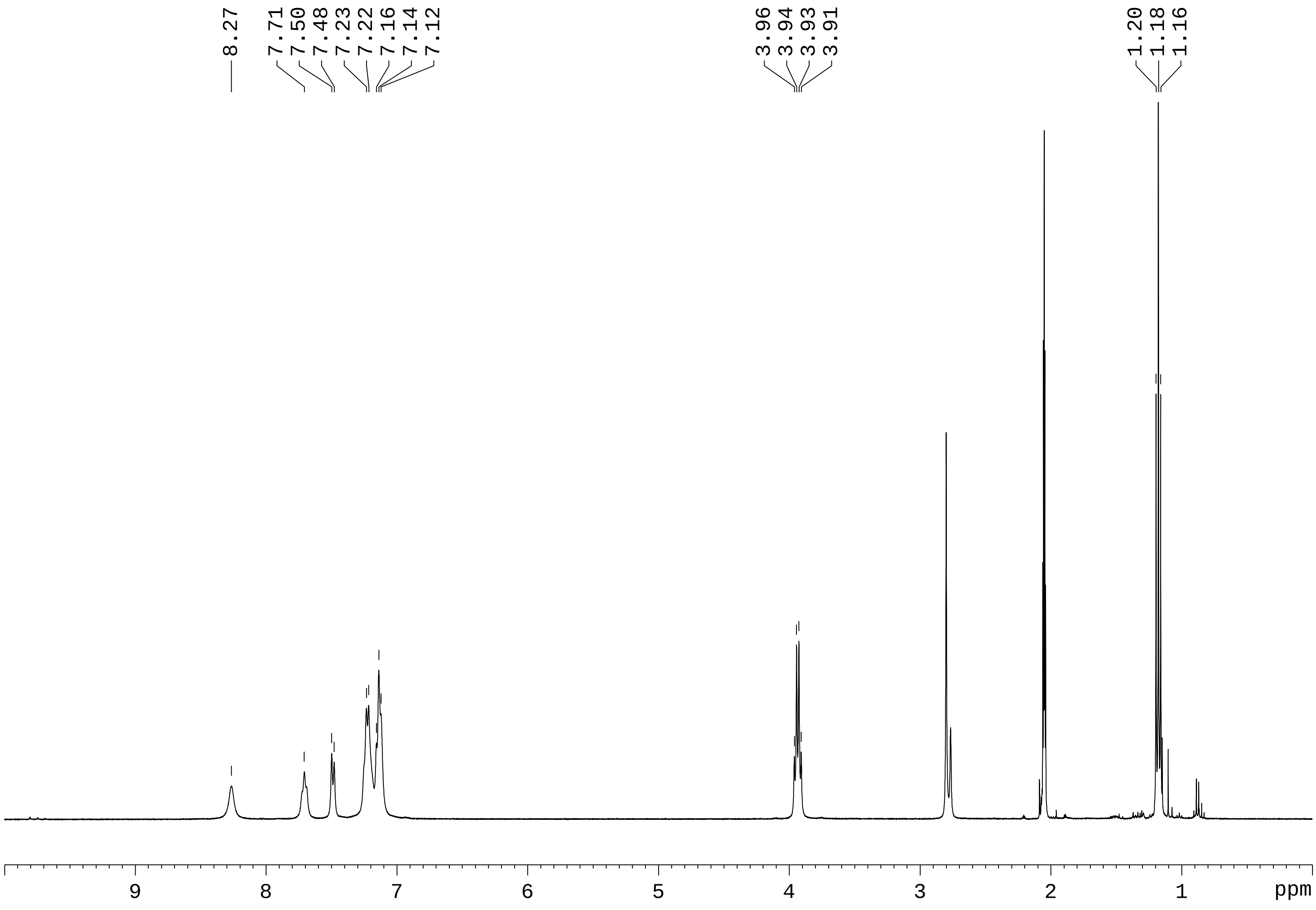

^{13}C NMR (100MHz, CD_3COCD_3) of *N*-ethyl-*N*-phenylpicolinamide **5b**

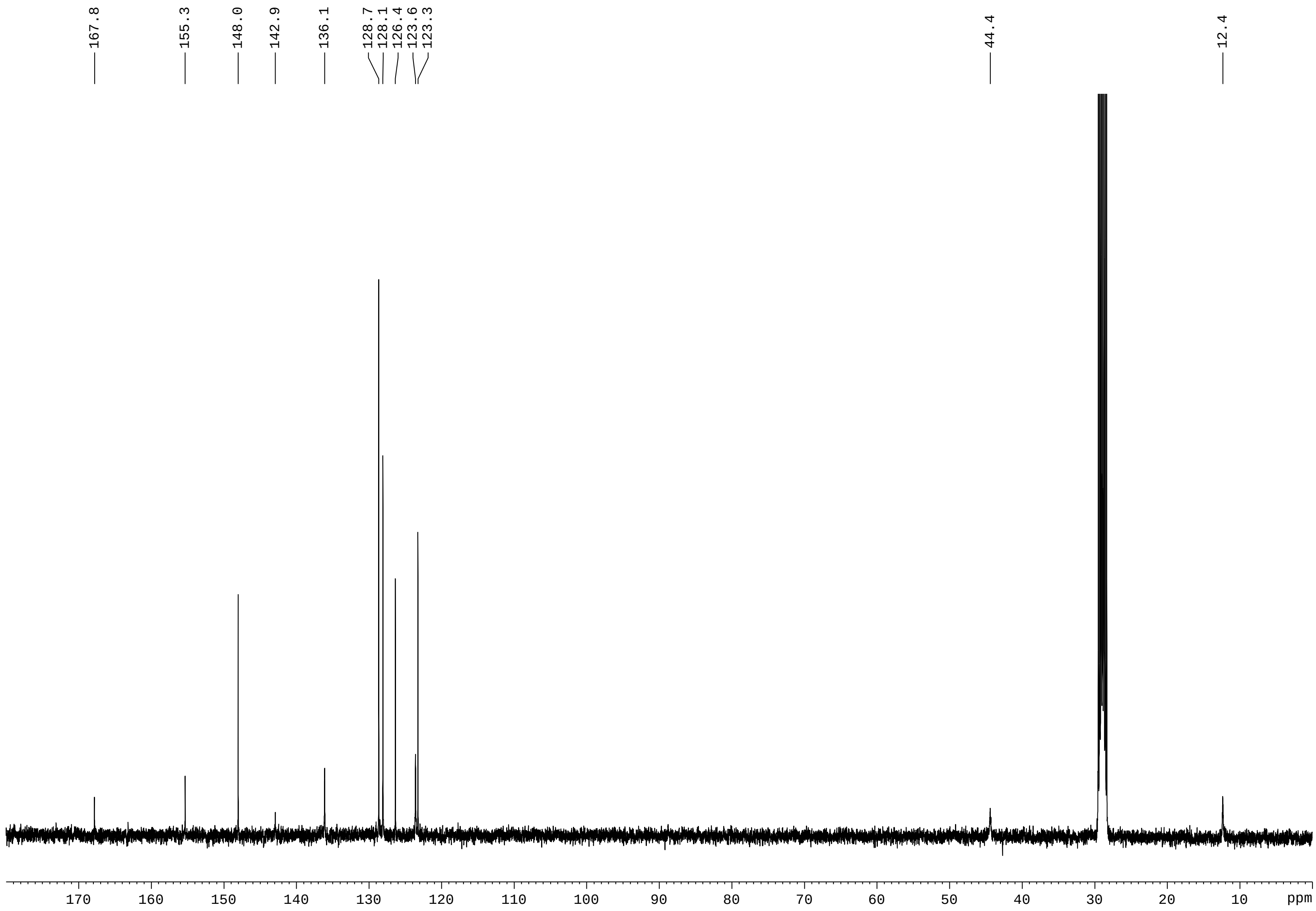

^{1}H NMR (400MHz, CD_3COCD_3) of N, N-diphenylpicolinamide **5c**

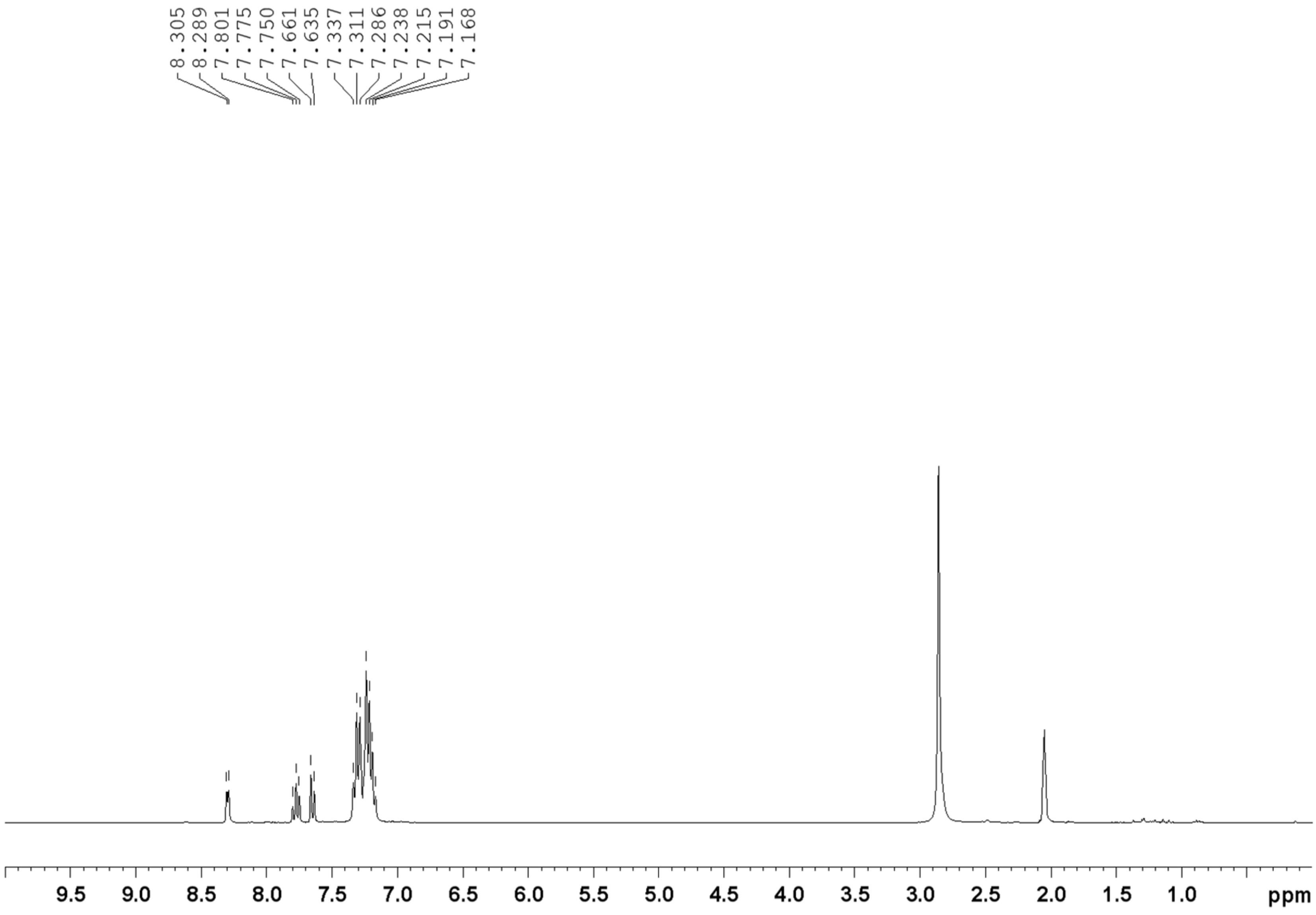

*^{13}C NMR (100MHz, CD_3COCD_3) of N, N-diphenylpicolinamide **5c***

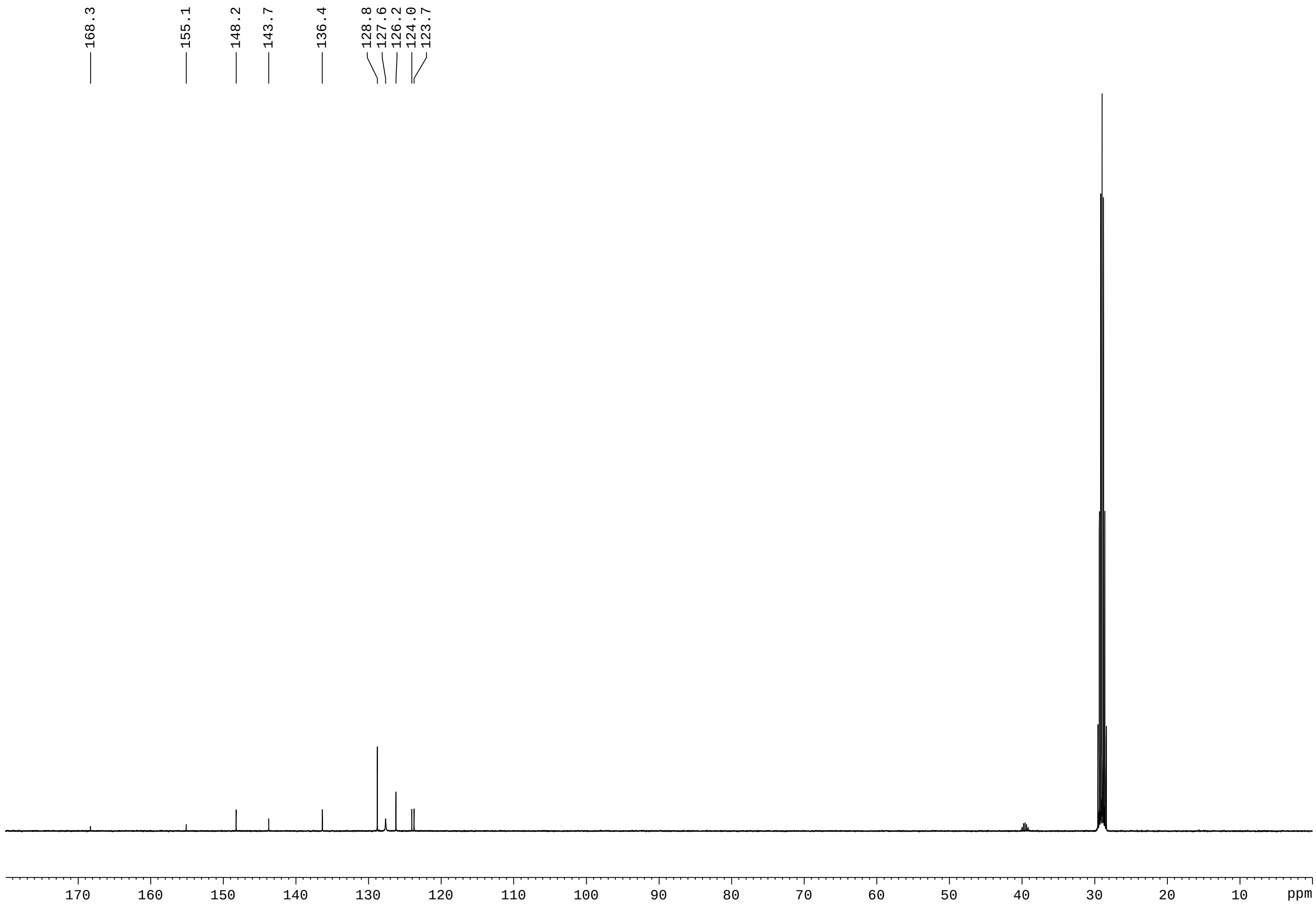

^{1}H NMR (400MHz, CD_3COCD_3) of 4-chloro-N-methyl-N-phenylpicolinamide **6a**

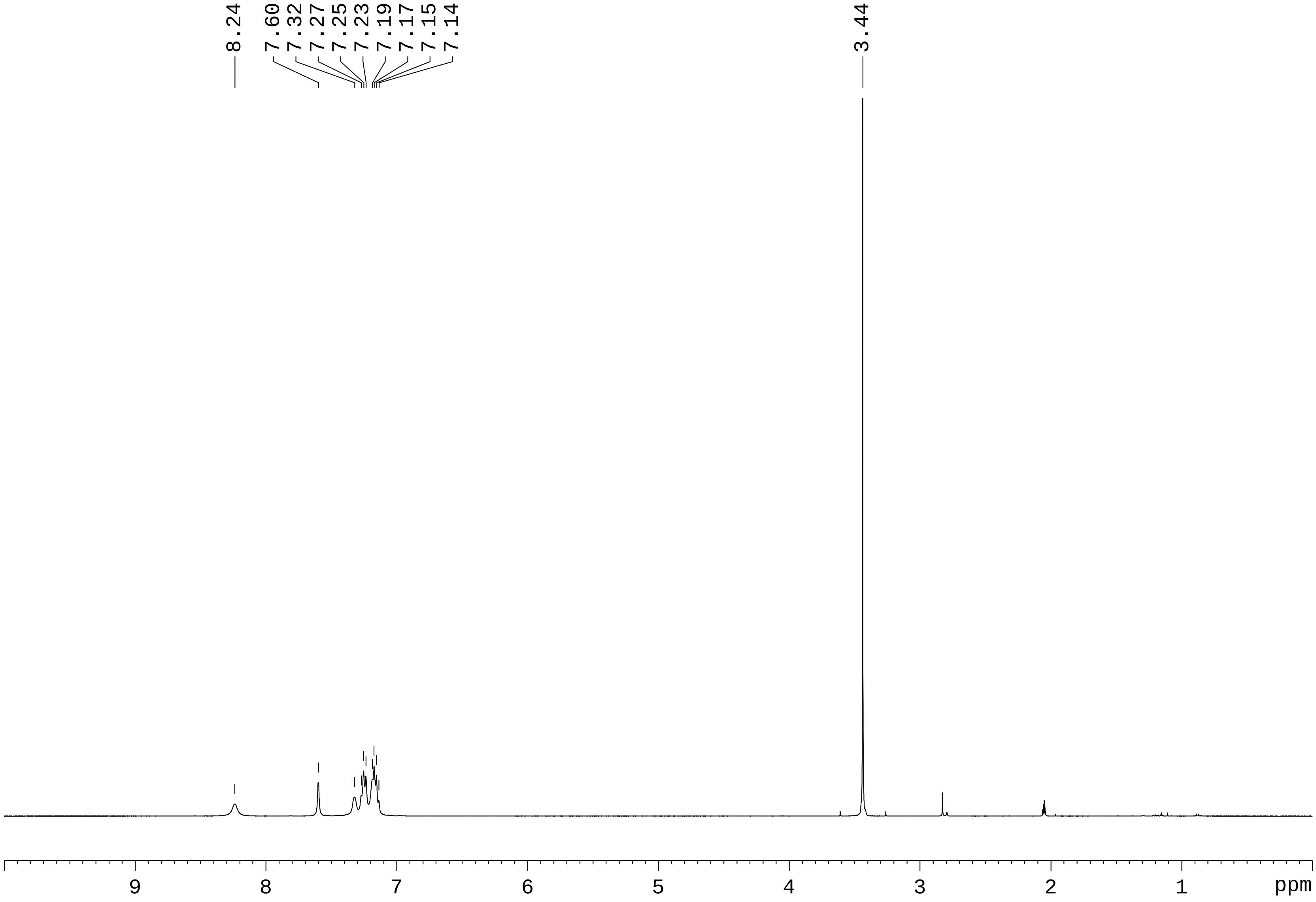

^{13}C NMR (100MHz, CD_3COCD_3) of 4-chloro-N-methyl-N-phenylpicolinamide **6a**

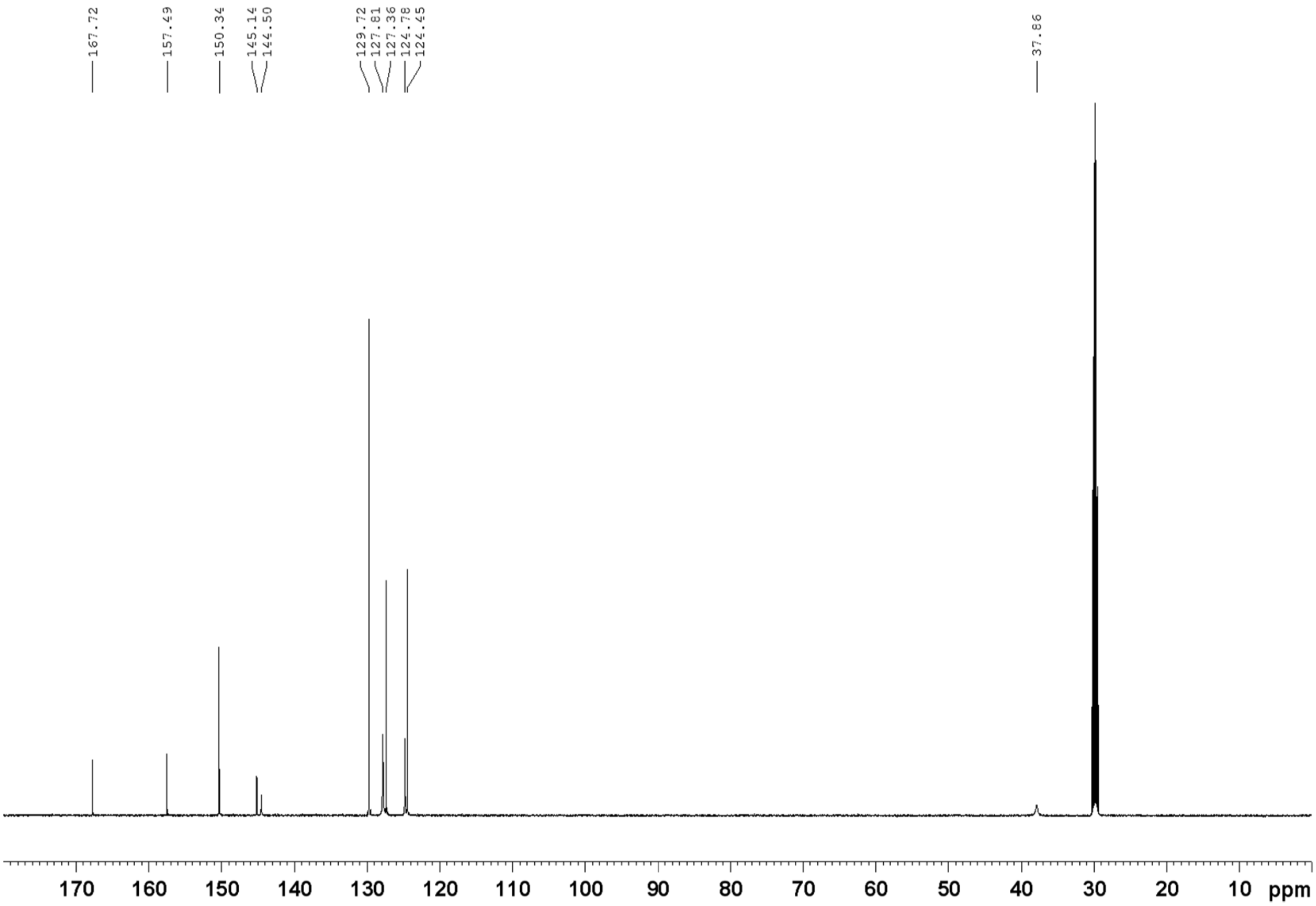

^{1}H NMR (400MHz, CD_3COCD_3) of 4-chloro-N-ethyl-N-phenylpicolinamide ***6b***

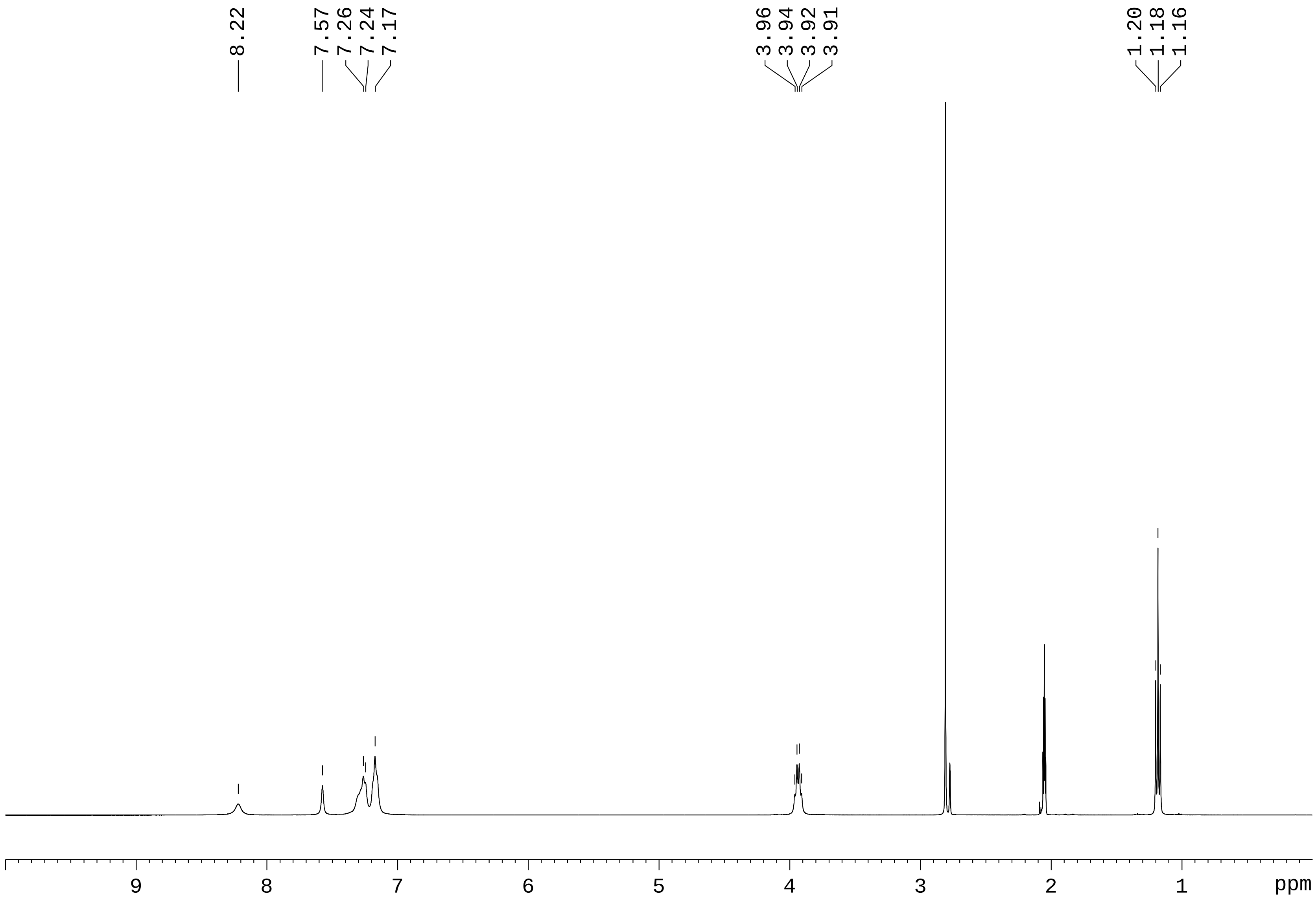

^{13}C NMR (100MHz, CD_3COCD_3) of 4-chloro-N-ethyl-N-phenylpicolinamide ***6b***

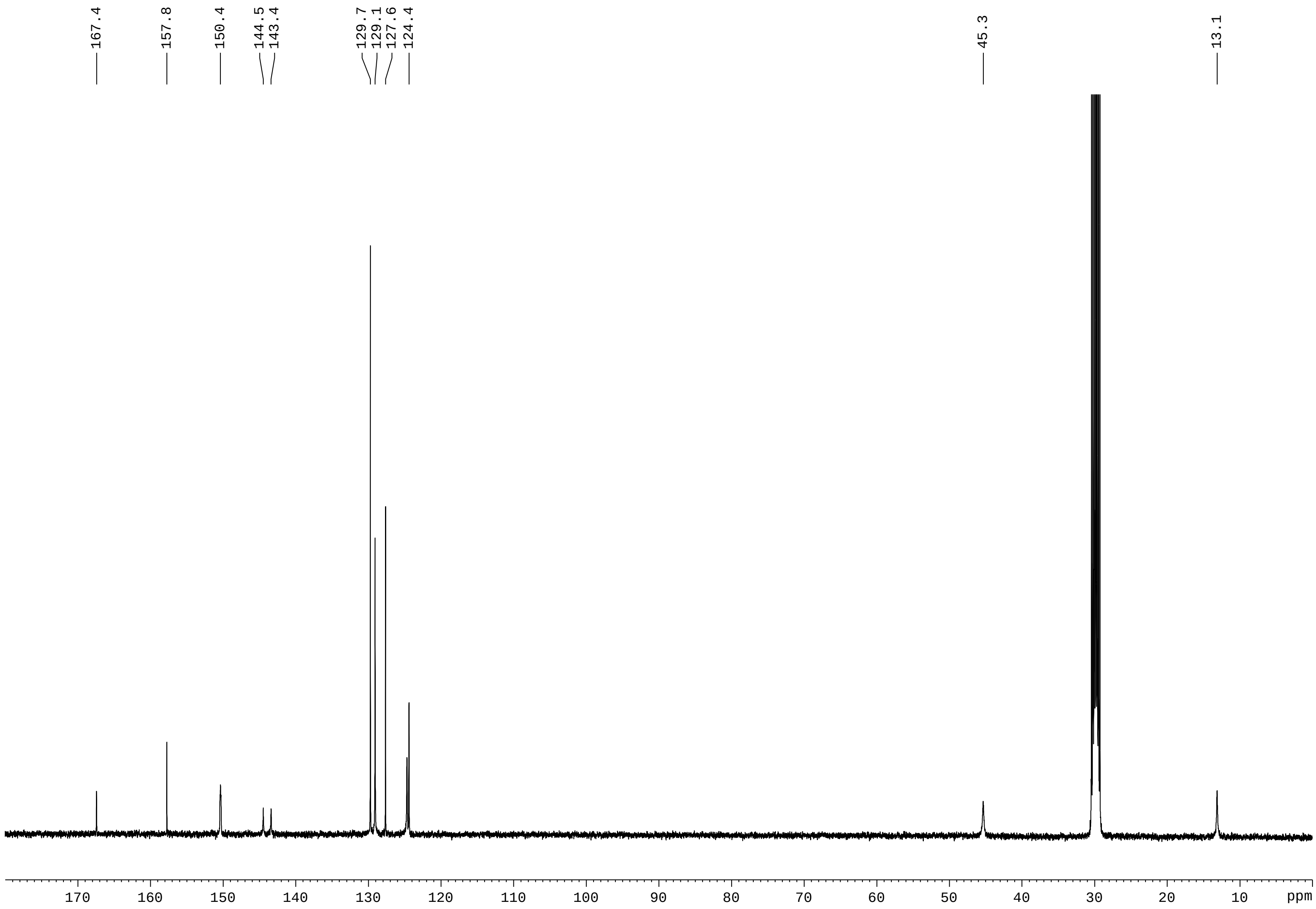

^{1}H NMR (400MHz, CD_3COCD_3) of 4-chloro-N, N-diphenylpicolinamide ***6c***

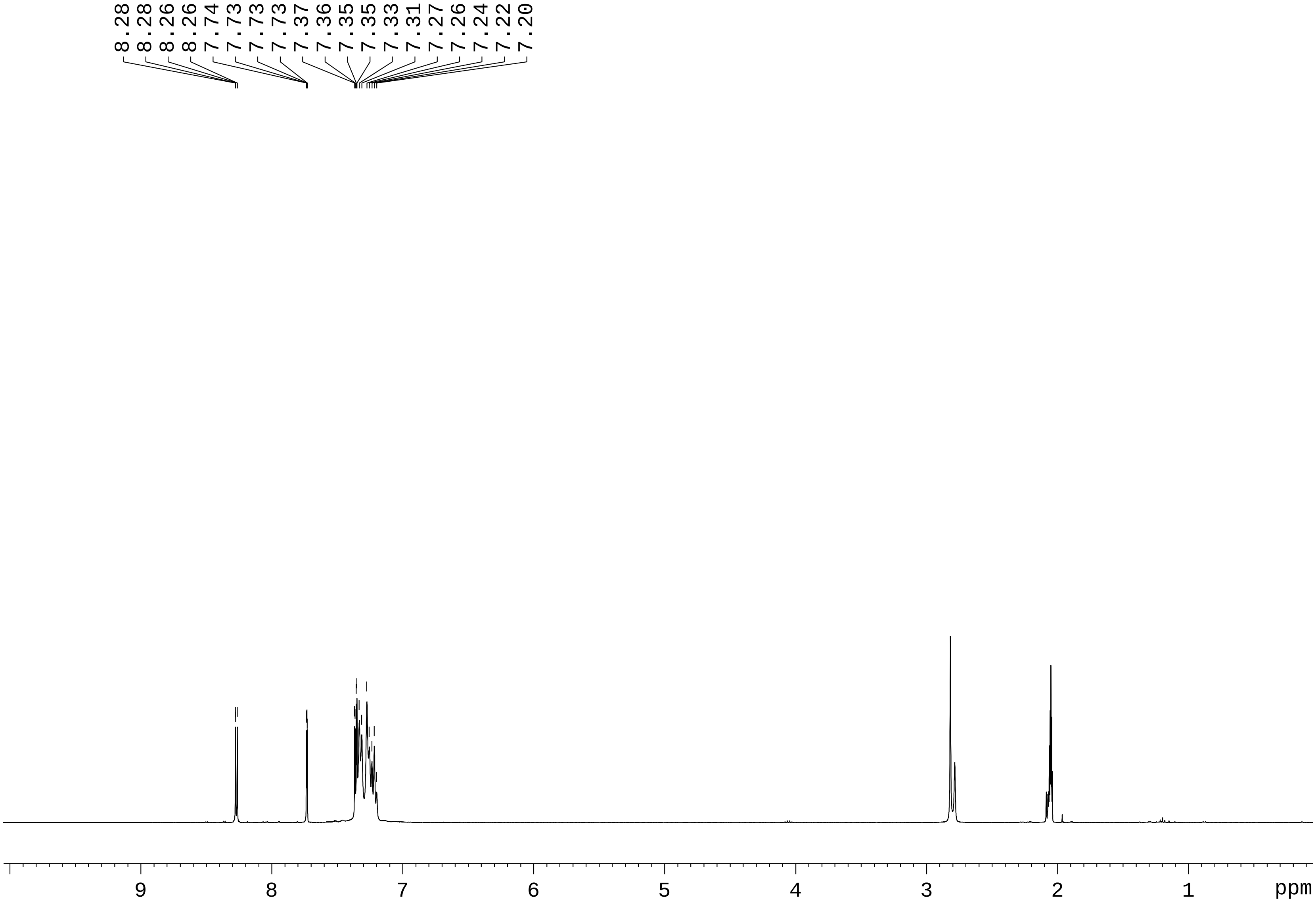

^{31}C NMR (100MHz, CD_3COCD_3) of 4-chloro-N, N-diphenylpicolinamide **6c**

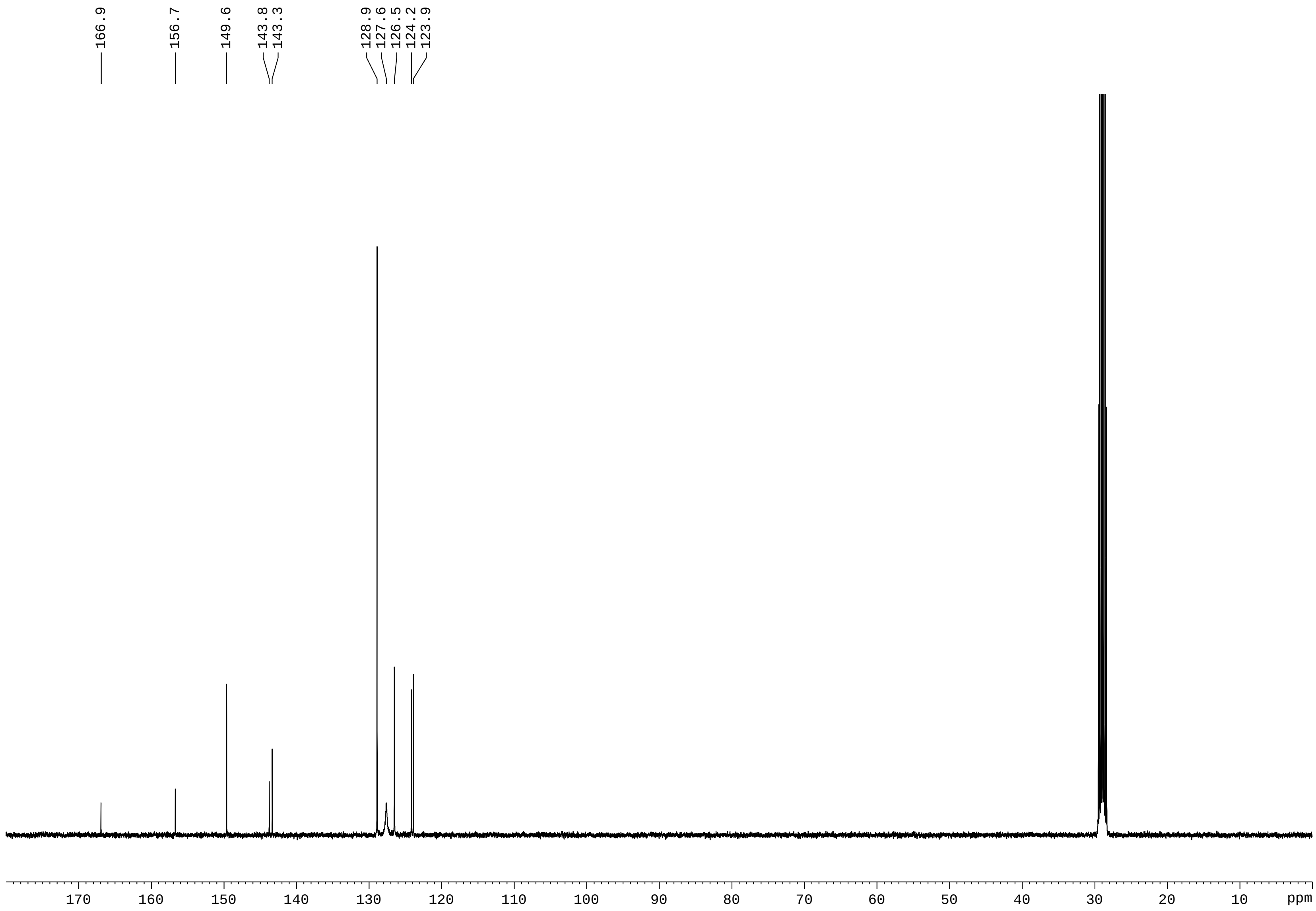

^{1}H NMR (400MHz, CD_3COCD_3) of N^2, N^6-dimethyl-N^2, N^6-diphenylpyridine-2, 6-dicarboxamide **7a**

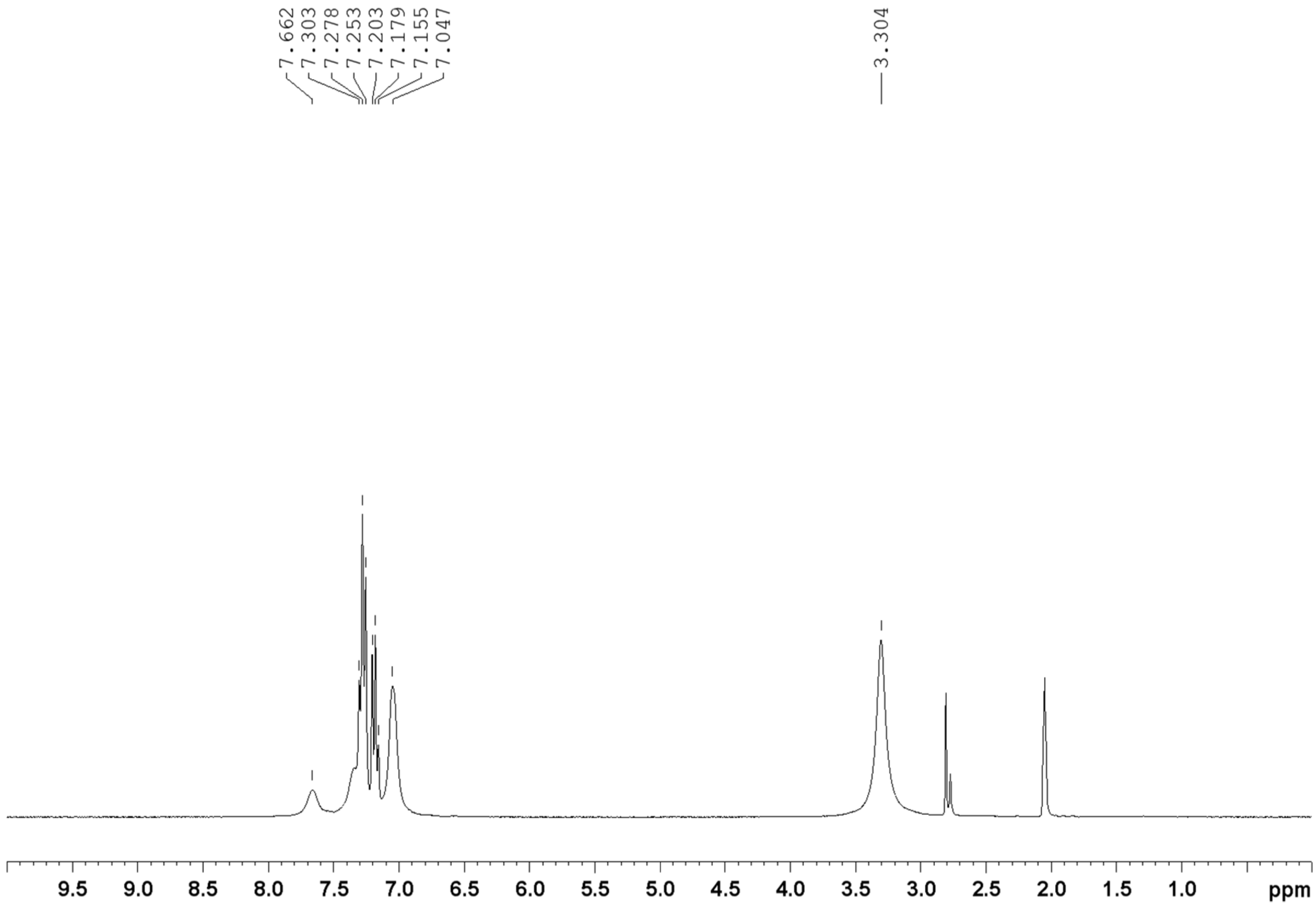

^{13}C NMR (100MHz, CD_3COCD_3) of N^2, N^6-dimethyl-N^2, N^6-diphenylpyridine-2, 6-dicarboxamide ***7a***

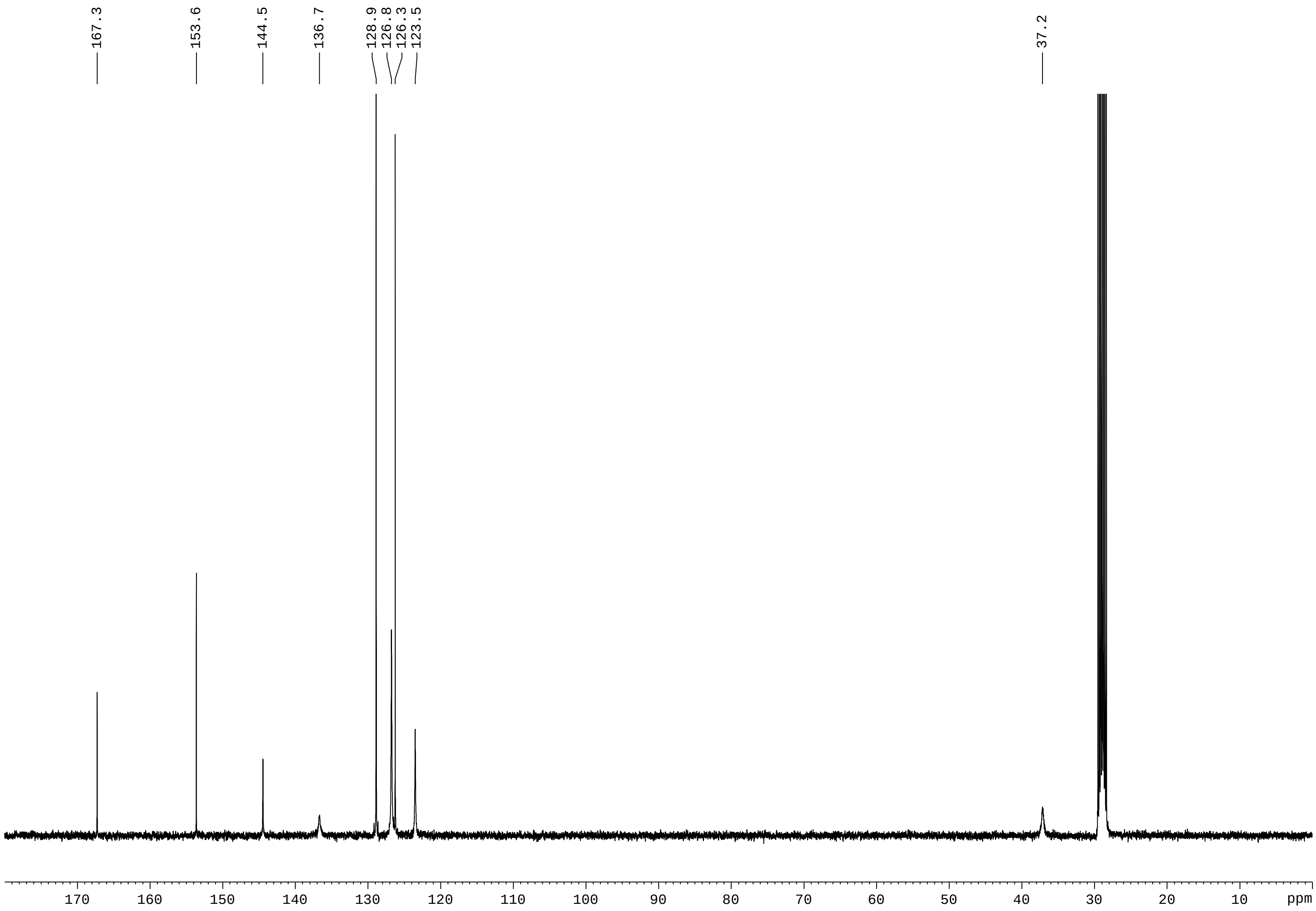

^{1}H *NMR (400MHz, CD_3COCD_3) of N^2, N^6-diethyl-N^2, N^6-diphenylpyridine-2, 6-dicarboxamide* ***7b***

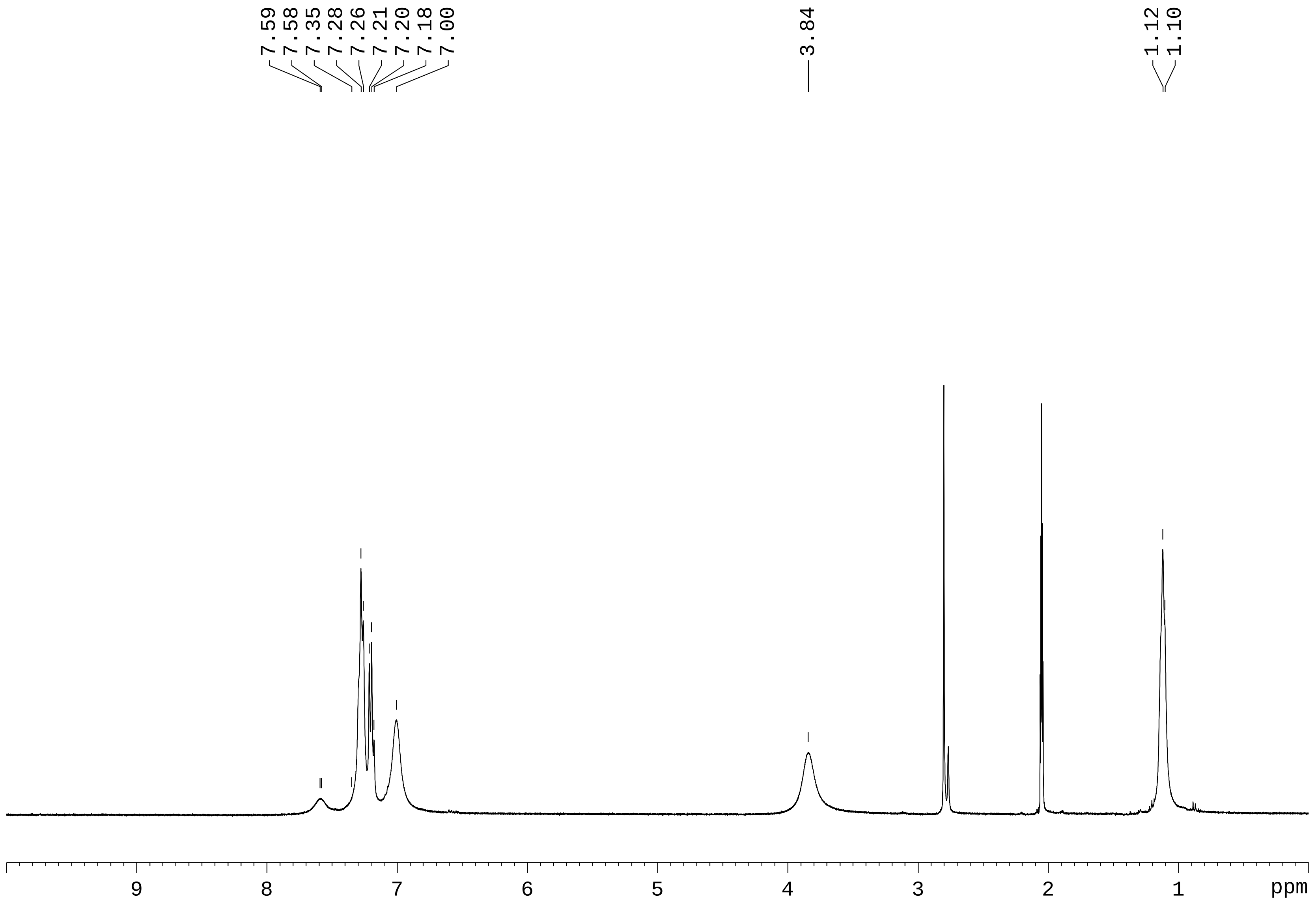

^{13}C NMR (400MHz, CD_3COCD_3) of N^2, N^6-diethyl-N^2, N^6-diphenylpyridine-2, 6-dicarboxamide **7b**

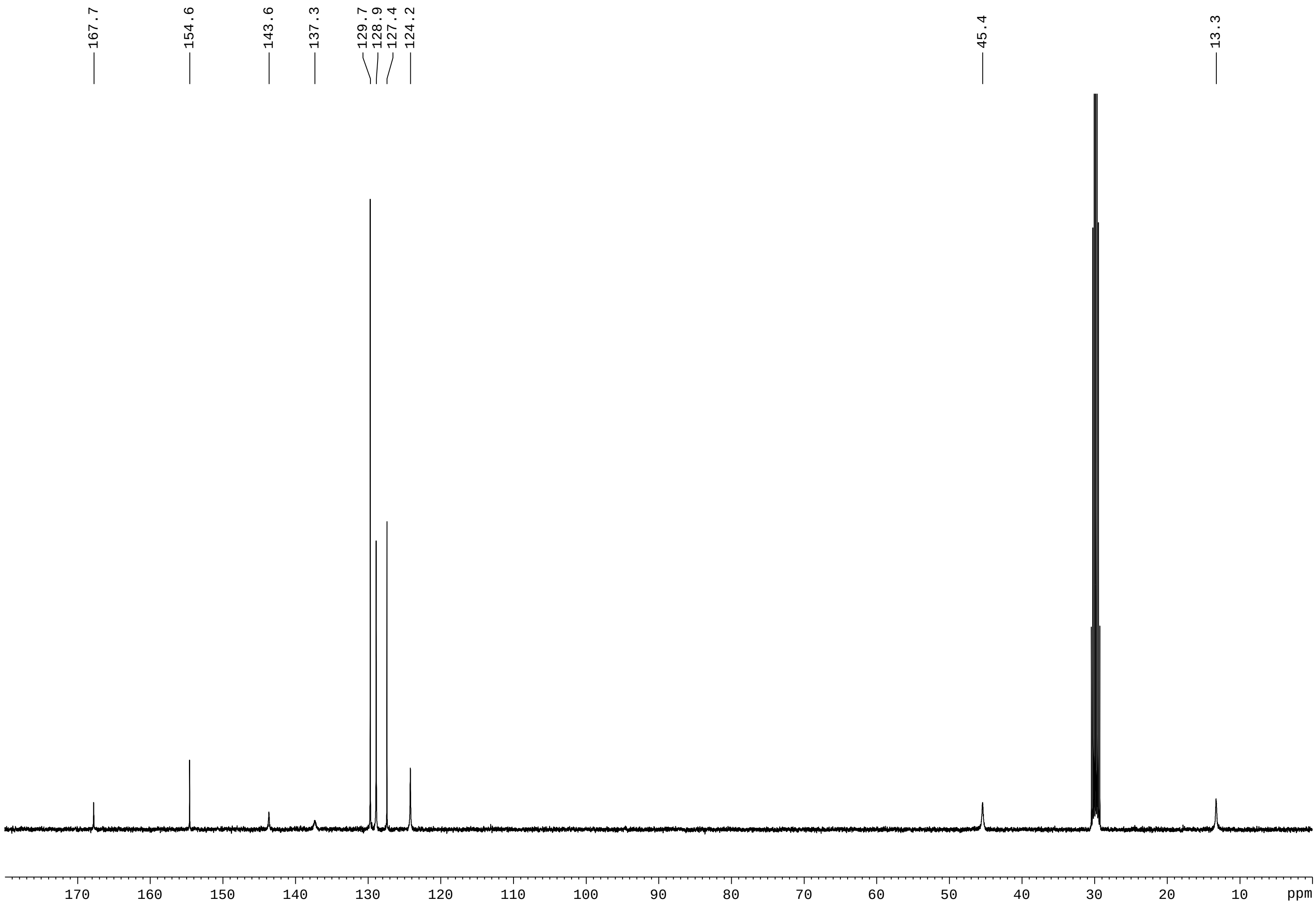

^{1}H NMR (400MHz, CD_3COCD_3) of N[2], N[2], N[6], N[6]-tetraphenylpyridine-2,6-dicarboxamide ***7c***

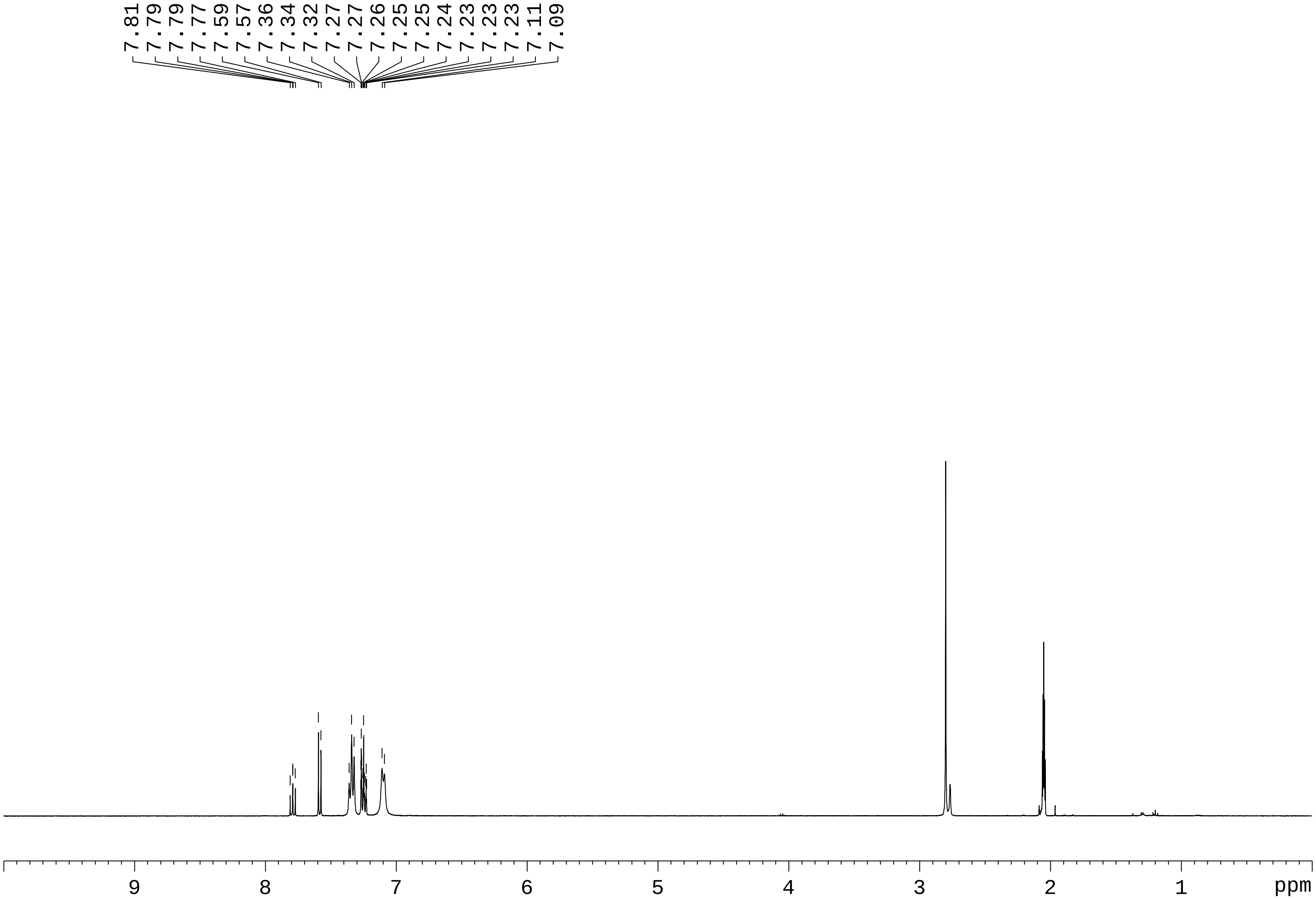

^{13}C NMR (100MHz, CD_3COCD_3) of N^2, N^2, N^6, N^6-tetraphenylpyridine-2,6-dicarboxamide **7c**

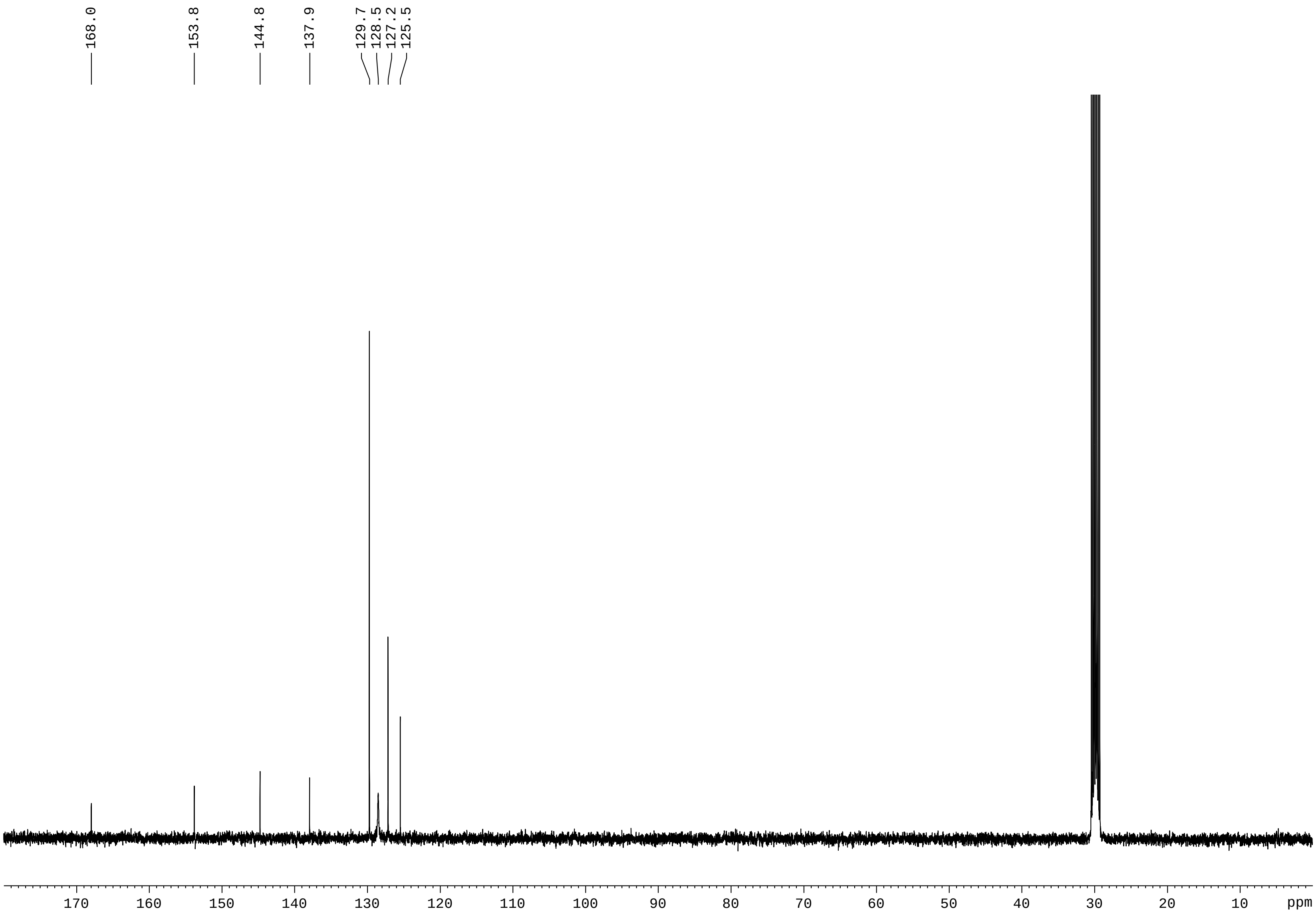

^{1}H NMR (300 MHz, $CDCl_3$) of Pyridine-2,6-dicarboxylic acid bis(L-valinyl-S-benzyl-L-cysteine methyl ester)carboxamide ***8a***

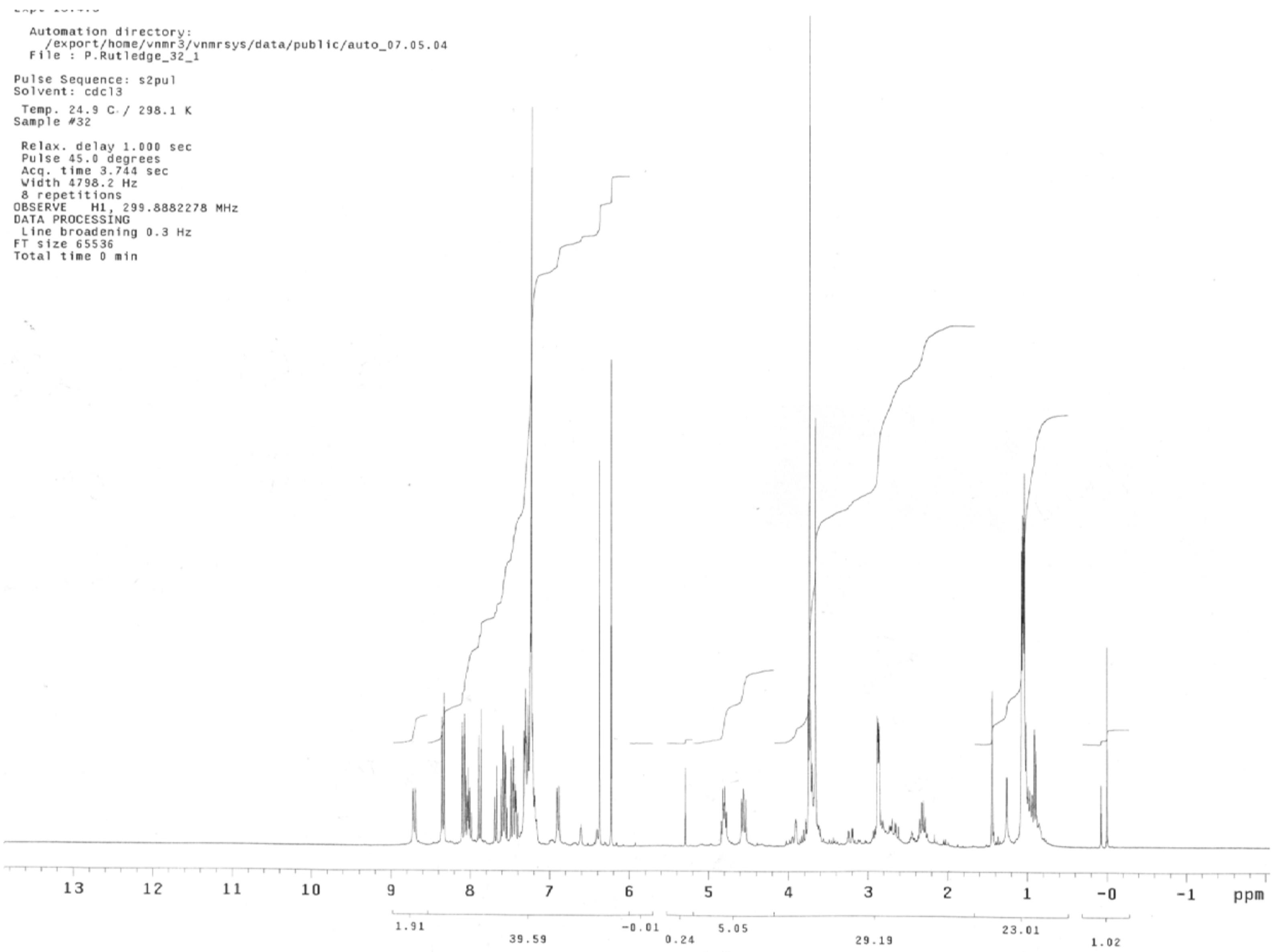

^{13}C NMR (75.4 MHz, $CDCl_3$) of Pyridine-2,6-dicarboxylic acid bis(L-valinyl-S-benzyl-L-cysteine methyl ester)carboxamide ***8a***

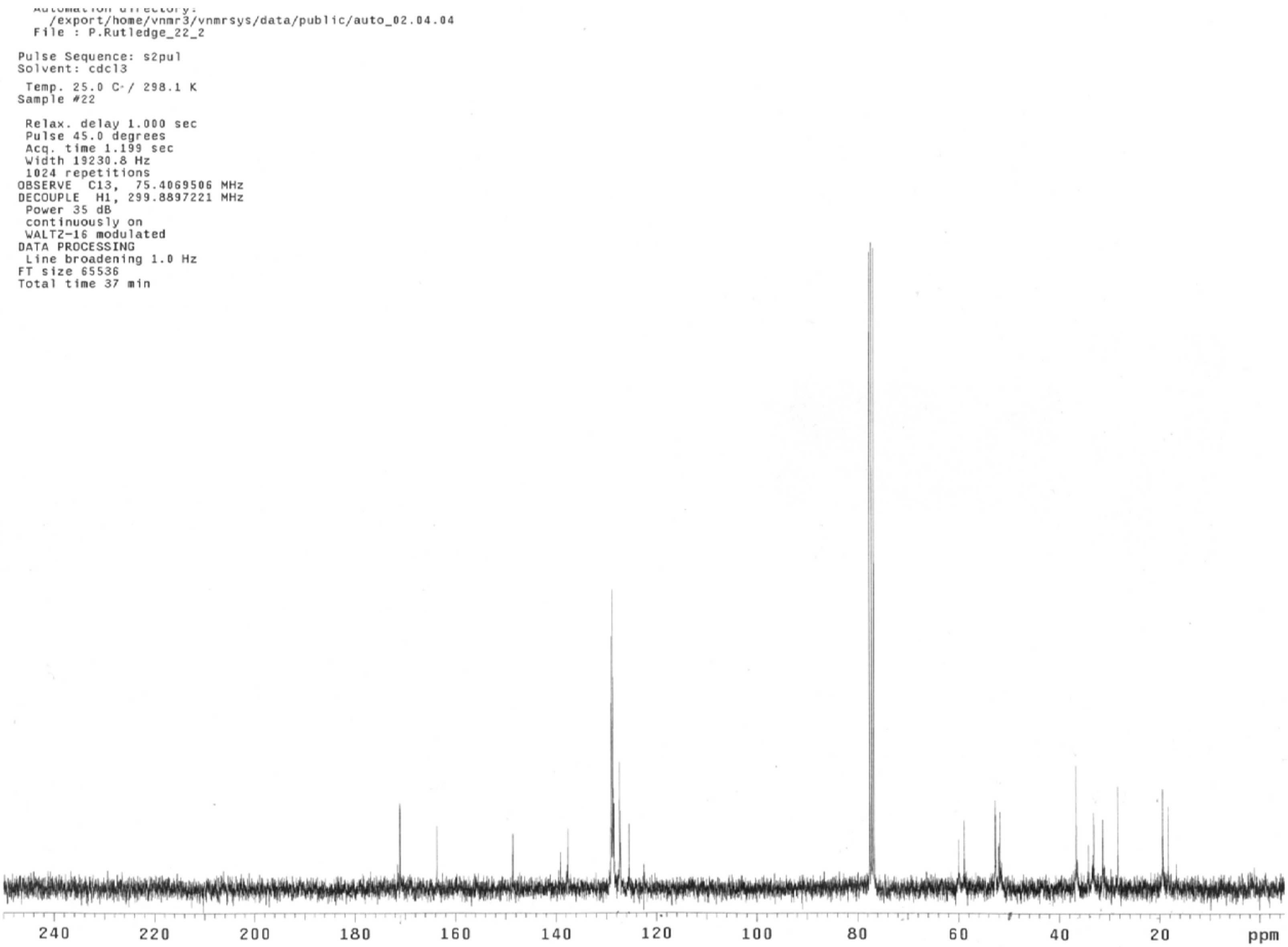

*^{1}H NMR (300 MHz, $CDCl_3$) of Pyridine-2,6-dicarboxylic acid bis(S-benzyl-L-cysteinyl-L-valine methyl ester)carboxamide **8b***

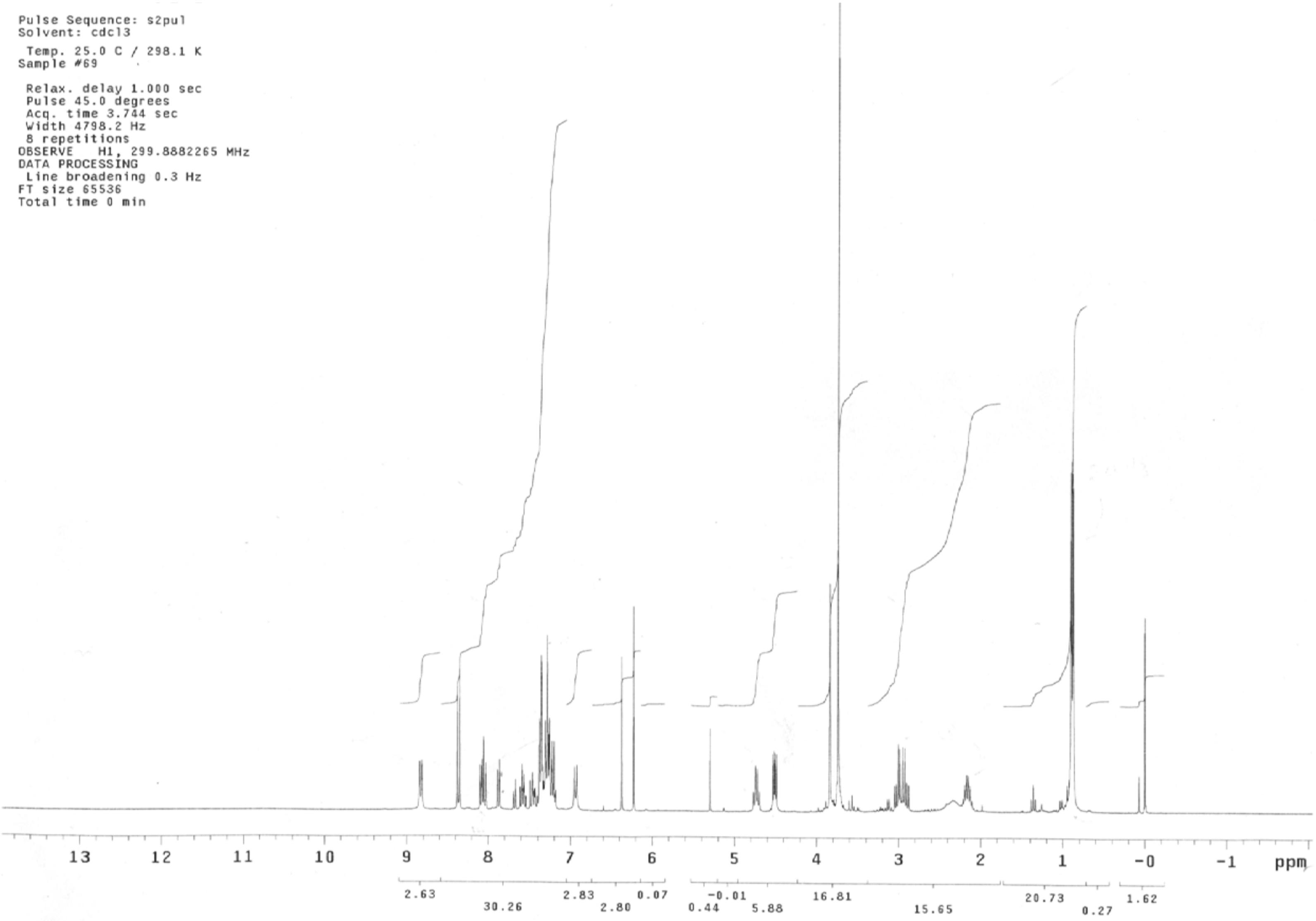

^{13}*C NMR (75.4 MHz, CDCl$_3$) of Pyridine-2,6-dicarboxylic acid bis(S-benzyl-L-cysteinyl-L-valine methyl ester)carboxamide* ***8b***

Expt 20.3
pad=2 run with findz0 before acquisition

Automation directory:
/export/home/vnmr2/vnmrsys/data/public/auto_06.10.04
File : P.Rutledge_04_01

Pulse Sequence: s2pul
Solvent: cdcl3

Temp. 25.0 C / 298.1 K
Sample #4

Relax. delay 1.000 sec
Pulse 45.0 degrees
Acq. time 1.199 sec
Width 19230.8 Hz
1024 repetitions
OBSERVE C13, 75.4069535 MHz
DECOUPLE H1, 299.8897221 MHz
Power 36 dB
continuously on
WALTZ-16 modulated
DATA PROCESSING
Line broadening 1.0 Hz
FT size 65536
Total time 37 min

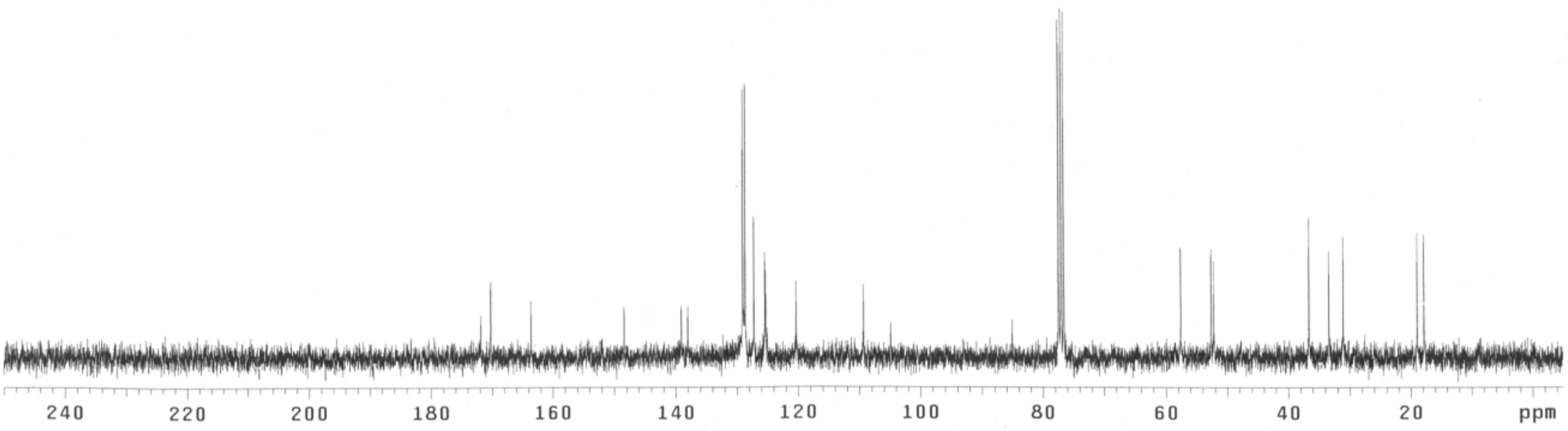

Supplement: Supplementary Information [file srep09950-s1.pdf]
